# PEG-Based Living Hydrogels Engineered for Tunable Production of Bioactive Lipopeptides

**DOI:** 10.1021/acsapm.6c00761

**Published:** 2026-06-30

**Authors:** Jeffrey A. Reed, Moises M. Gutierrez, Douglas A. Dougherty, Asad Bin Zaman, Ryan R. Hansen

**Affiliations:** † Tim Taylor Department of Chemical Engineering, 5308 Kansas State University, Manhattan, Kansas 66506, United States; ‡ Department of Chemical and Materials Engineering, 4423 New Mexico State University, Las Cruces, New Mexico 88003, United States

**Keywords:** hydrogels, engineered living materials, bacteria, surfactin, poly(ethylene glycol), wound dressings, biotherapeutics

## Abstract

Encapsulation of microbial cells within nanoporous hydrogels creates dynamic and responsive living materials well-suited for biotherapeutic applications. *Bacillus subtilis* is a promising microbe in this application as it is generally regarded as safe, can be sporulated for long-term stability and resistance to nonideal environments, and can produce antimicrobial and anticancer molecules such as the cyclic lipopeptide surfactin. Here, we examine the growth of *B. subtilis* cells and corresponding production of surfactin after encapsulation within poly­(ethylene glycol) (PEG) hydrogels at varied levels of nanoconfinement. Encapsulation was achieved through Michael-type addition reactions between PEG diacrylate and PEG tetrathiol macromers, where macromer molecular weight was systematically varied to generate hydrogels across a range of average mesh sizes (9–19 nm). Hydrogels had varied Young’s modulus (7.3 ± 1.7 kPa to 16.4 ± 0.7 kPa) and provided a 7-fold range in small molecule diffusivity. In-situ cellular growth monitoring and surfactin quantification revealed that all hydrogels stimulated the production of surfactin with verified antibacterial activity and in a manner tunable with mesh size. Smallest mesh sizes drove highest surfactin production, a ∼5-fold increase relative to equivalent cultures of unconfined cells. Cell loading was then varied in 9 nm mesh size hydrogels to reveal that low cell loading (0.1–1 × 10^4^ cells per μL hydrogel) promoted sustained growth and surfactin production proportional to the number of cells loaded. Conversely, hydrogels loaded with excessive cells (2.5–7.5 × 10^4^ cells per μL hydrogel) resulted in unsustained growth and diminished surfactin production. Finally, to develop a more robust material, *B. subtilis* endospores were encapsulated into hydrogels at optimized conditions. Spore-laden hydrogels retained the capability to produce surfactin after exposure to dehydration and temperature stress. These results indicate that hydrogel encapsulation stimulates *B. subtilis* surfactin production according to the level of nanoconfinement to achieve a tunable engineered living material for production of bioactive molecules.

## Graphical abstract

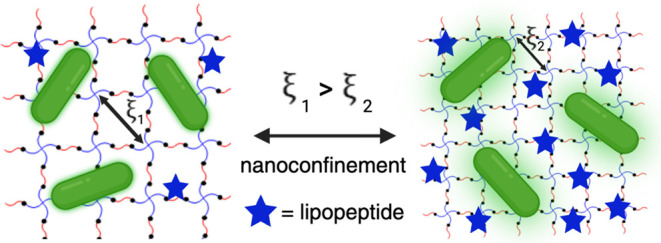

## 1 Introduction

Engineered living materials (ELMs) are formed by combining live cells with compatible biomaterials to enable a broad range of biomedical applications, including in-situ production and delivery of biotherapeutic molecules for wound healing and anticancer applications.^1^ Due to their diverse functionality, programmable nature, and relative simplicity, bacteria have received extensive interest as the cellular component in these applications,^2,3^ while hydrogels are often the biomaterial of choice because they encapsulate cells to control their spatial distribution while providing a microenvironment conducive to their survival, proliferation, and function.^4,5^ In wound healing applications, bacteria encapsulated within hydrogels can produce therapeutic molecules (e.g., antibiotics) in response to environmental cues such as pH, hypoxia, and reactive oxygen species to treat infection sites.^6,7^ Similarly, in anticancer applications, hypoxic conditions created by cancer cells can be sensed by bacteria in hydrogels for production of molecules cytotoxic to the tumor environment.^8^ While naturally occurring, polysaccharide-based hydrogels (alginate, chitosan, hyaluronic acid) are most commonly used,^9^ synthetic hydrogels such as poly­(ethylene glycol) (PEG), poly­(acrylic acid) (PAA), and poly­(vinyl alcohol) (PVA) offer distinct design advantages, including precise control of physicochemical hydrogel properties,^10^ long-term stability,^11^ or tunable hydrogel degradation.^12^

Use of PEG in ELM applications is particularly advantageous because PEG is chemically and biologically inert and resistant to cellular adhesion and biomolecule adsorption.^13–15^ These features yield PEG-based hydrogels that resist external bacterial colonization and biofilm formation while promoting the function of encapsulated bacteria, a critical feature for wound healing applications.^16^ Further, PEG-based macromers used in hydrogel precursor solutions are commercially available across a wide variety of molecular weights and end-group chemistries and can be modified to incorporate stimuli-responsive moieties,^17^ enabling flexible and creative hydrogel design options tailored toward specific applications.^18^ In particular, the molecular weight of the PEG precursors can be varied to tune hydrogel average mesh size (ξ) for control of mass transport, particularly in step-growth hydrogels that yield high mesh size uniformity.^19^ Mesh size also determines hydrogel mechanical properties, equilibrium swelling, and the degree of physical confinement imposed on encapsulated cells, with smaller mesh sizes corresponding to lower swelling, stiffer gels, and greater diffusion limitations.^20^ These factors influence how cells regulate gene expression, growth, and morphology. While such behaviors are well studied with encapsulated eukaryotic cells for tissue engineering applications,^21^ similar studies with microbial cells for emerging ELM applications are currently underexplored.^4^

The primary goals of this study are to understand the effects that hydrogel nanoconfinement, as determined by hydrogel mesh size, and *Bacillus subtilis* loading play in cellular growth and corresponding production of the secondary metabolite, surfactin. *B. subtilis* has generally recognized as safe (GRAS) designation and the unique ability to form endospores, decisive advantages for real-world ELM applications. *B. subtilis* strain 21332 was chosen here, as it produces bioactive lipopeptide molecules (e.g., surfactin, fengycin) with potent antibacterial, antifungal, and anticancer properties,^22,23^ and is the “gold-standard” surfactin-producing strain among nongenetically modified *B. subtilis* organisms.^24^ Surfactin has been demonstrated to prevent the growth of pathogenic biofilms and decrease wound closure times,^25^ while also inducing apoptosis in human breast cancer cells.^26^ For example, previous work by David et al. demonstrated the encapsulation of *B. subtilis* within PVA microparticles for producing surfactin at 350 μg/mL concentrations to treat both *Staphylococcus aureus* and methicillin-resistant *S. aureus*.^27^ Here, we encapsulate *B. subtilis* in hydrogels of varied mesh sizes using Michael-type addition reactions between PEG diacrylate (PEGDA) and PEG tetrathiol (PEGTT) macromers across a range of macromer molecular weights (Figure 1). This encapsulation chemistry was previously demonstrated to immobilize bacteria cells at high viability,^28^ generating hydrogels that facilitate bacteria growth,^29–31^ and that also provide controlled transport and release of bacteria on degradation.^32–34^

**Figure 1:**
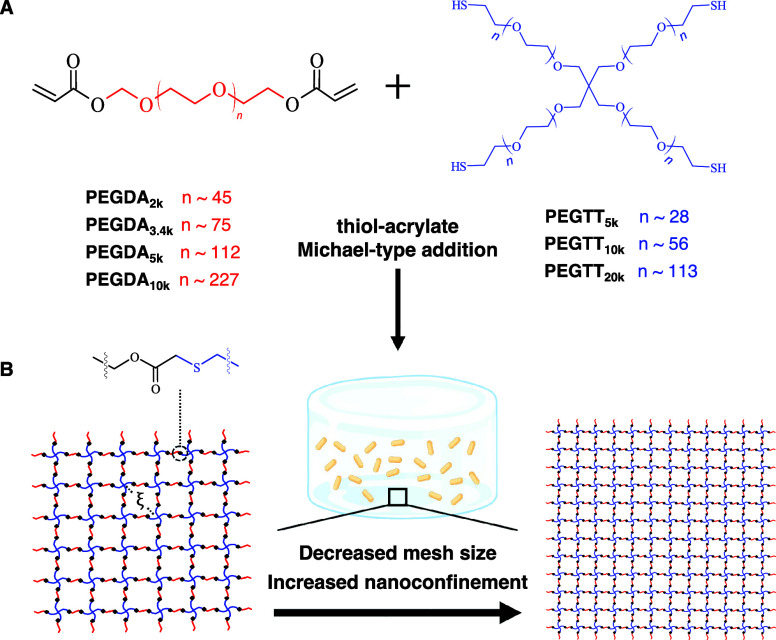
(A) Formation of PEG hydrogels through Michael-type addition reaction between PEGDA and PEGTT macromers of varied molecular weights. (B) Decreasing mesh sizes (ξ) increase nanoconfinement on encapsulated bacteria. Panel (B) was partially completed in BioRender software.

The results reveal that hydrogel mesh size (ξ = 9–19 nm) can be used to provide *B. subtilis* with varied levels of nanoscale physical confinement to enhance the quorum-mediated production of surfactin. Production was tunable with mesh size, with the lowest mesh size and highest nanoconfinement generating the highest surfactin production. Variation of *B. subtilis* cell loading levels demonstrated that low cell loading provides surfactin levels proportional to the number of cells added, while overloading hydrogels with cells diminishes growth and surfactin production. Finally, using hydrogels designed for maximum surfactin production, we encapsulate *B. subtilis* endospores and demonstrate the ability to retain surfactin production after exposing hydrogels to dehydration and temperature stress. These combined results will inform the future design of PEG hydrogels in ELM applications that include living bandages, wound dressings, and anticancer therapies.

## 2 Materials and Methods

### 2.1 Material Preparations

Four separate PEGDA macromers of different molecular weights (*M*_n_ = 2, 3.4, 5, and 10 kDa) were purchased from Creative PEGWorks, and three separate PEGTT macromers of different molecular weights (*M*_n_ = 5, 10, and 20 kDa) were purchased from NOF America. *B. subtilis* strain 21332 was obtained from the American Type Culture Collection (ATCC), and *B. subtilis* strain 168 was obtained from the Bacillus Genetic Stock Center (Strain 1A1135). *Escherichia coli* strain K12 was gifted from Oak Ridge National Laboratory. Stocks of each strain were maintained in a 50% glycol solution and stored at −80 °C. Nutrient broth, buffer salts, and other reagents were purchased from Sigma-Aldrich unless otherwise noted. Phosphate-buffered saline (PBS) buffer at 0.1 M was made in-house by dissolving 1,567 mg NaH_2_PO_4_ and 187 mg Na_2_EDTA in 90 mL ultrapure water and adjusting the pH to 8.0 with aqueous 5 M NaOH. The PBS buffer was sterile-filtered and stored at room temperature prior to use. ATGN-phosphate buffer was prepared by combining 1,506 mg of sodium phosphate monobasic dihydrate with 95 mL of 2 × AT minimal medium, supplemented with glucose (Fisher Scientific) and ammonium sulfate ((NH_4_)_2_SO_4_, Fisher Scientific), as described by Morton and Fuqua.^35^ The pH of the solution was then adjusted to 8.0 using an aqueous 5 M sodium hydroxide solution (Fisher Scientific), sterile filtered, and stored at −20 °C until further use. 2 × Schaffers glucose (2 × SG) medium from Leighton and Doi^36^ was used for *B. subtilis* endospore formation. 2 × SG media was made by combining 16 g per liter nutrient both No. 4 with 0.5 g per liter MgSO_4_·7 H_2_O (Macron Fine Chemicals), 2.0 g per liter KCl (Fisher Scientific), and autoclaved. After autoclaving, the medium was supplemented with 1 mM Ca­(NO_3_)_2_ (Fisher Scientific), 0.01 mM MnCl_2_, 0.001 mM FeSO_4_ (added as FeSO_4_·7 H_2_O, Wards Science), and 0.1% (w/v) glucose. All growth experiments with bacteria encapsulated within a hydrogel used M9 minimal media unless otherwise noted. M9 minimal media was made according to Miller,^37^ combining 6 g/L Na_2_HPO_4_, 3.0 g/L KH_2_PO_4_, 0.5 g/L NaCl (Fisher Scientific), 1.0 g/L NH_4_Cl (Fisher Scientific), and autoclaving. Post autoclave additions included 10 mL of 100 mM MgSO_4_, 20% glucose, 10 mM CaCl_2_ (Fisher Scientific), and 100 mM thiamine HCl. Tryptic soy broth (TSB) was made according to the manufacturer’s instructions.

### 2.2 Hydrogel Preparation and Mesh Size Determination

Hydrogels of various mesh sizes were formed via Michael-type addition reaction on mixing of an aqueous solution of PEGDA macromer with an aqueous solution of PEGTT macromer. PEGDA macromers were reacted pairwise with the PEGTT macromers in varied molecular weight combinations to create a total of 11 different macromer combinations, generating hydrogels of 11 different mesh sizes. Hydrogels were formed at either 25, 50, 100, or 200 μL volumes. For 100 μL hydrogel volumes, 50 μL PBS was mixed with 22.4 μL of PEGDA (49 mM) dissolved in Milli-Q water. After thorough mixing, 27.6 μL of PEGTT (20 mM) dissolved in Milli-Q water was then added, achieving an equimolar ratio of acrylate and thiol groups. Volumes were scaled accordingly for 25, 50, or 200 μL hydrogels. Each mixture was reacted at room temperature for 30 min for hydrogel formation.

For hydrogel swelling experiments, hydrogels were prepared at 200 μL, then fully swollen or dried, weighed, and Equilibrium Swelling Theory as described by Canal and Peppas was used to calculate average mesh size for each hydrogel.^38^ For equilibrium swelling, 500 μL of water was pipetted on top of a hydrogel in a 2 mL vial and incubated for 24 h. For drying, 200 μL hydrogels were placed in a 2 mL vial in an oven at 80 °C for 24 h. After swelling or drying, hydrogel samples were removed from their respective vials and weighed with a digital analytical balance (U.S. Solid, ±0.1 mg precision). A summary of the equations and constants used for equilibrium swelling calculations is provided in the Supporting Information file (S1.0). All swollen and dry hydrogel masses were measured on n = 3 independent hydrogels for each macromer combination.

### 2.3 Diffusional and Mechanical Hydrogel Characterizations

Effective diffusion coefficients of Alexa Fluor 488 (D_eff_) through PEG hydrogels of varied mesh size were experimentally determined using a small molecule desorption approach adapted from previous hydrogel diffusion studies.^31^ Cylindrical 150 μL hydrogels were formed inside 2 mL glass vials and swollen in 1 mL ultrapure water for 24 h. The ultrapure water was removed, and the hydrogels were subsequently equilibrated with a 282 μM Alexa Fluor 488 solution for 24 h. Following loading, the Alexa Fluor solution was removed and replaced with 1.0 mL ultrapure water to initiate Alexa fluor diffusion out of the hydrogel and into solution. At selected time points, 2 μL aliquots of the release solution were collected at and analyzed using a Nanodrop One UV–Vis spectrophotometer at 494 nm. Alexa fluor concentrations were calculated using Beer’s law from standards prepared by diluting the original 282 μM Alexa fluor to varied concentrations. Released tracer concentrations were normalized to the equilibrium release concentration at the final time point (*C*_t_/*C*_∞_). Effective diffusion coefficients were then estimated using the early time approximation of Fick’s second law for slab geometries when $\frac{C_{t}}{C_{\infty }}\leq 0.5-0.6$ :^39^

$$\frac{C_{t}}{C_{\infty }}=4{\left(\frac{D_{eff}t}{\pi L^{2}}\right)}^{1/2}$$

where C_t_ and C_∞_ are the Alexa Fluor 488 concentrations measured at time *t* and at equilibrium, respectively, *L* is the swollen hydrogel thickness, and *D*_*eff*_ is the effective diffusion coefficient of Alexa Fluor 488 through the hydrogel. Swollen hydrogel thicknesses used for diffusion calculations were estimated from equilibrium swelling measurements and experimentally estimated via cross-sectional areas (S2.0, Table S1).

Hydrogel mechanical properties were evaluated by compression using an Instron universal testing system equipped with a 5 kN load cell and 3D printed flat plate cylinders (diameter = 16 mm, height = 25 mm) designed in AutoCAD. Hydrogels were formed as nominal 4 × 4 × 4 mm cubes by mold casting, pipetting 64 μL hydrogel precursor solutions into cubic molds of the same dimension (Figure S1). Following gelation, hydrogels were removed from the molds and immediately subjected to compression testing to minimize dehydration effects. Individual hydrogel cubes were positioned between the compression plates and compressed at a constant crosshead speed of 0.5 mm/min at room temperature. Force and displacement data were recorded every 0.01 s throughout testing. Compression was continued until the stress–strain response became nonlinear. Engineering stress was calculated using the nominal hydrogel cross-sectional area of 16 mm^2^, and engineering strain was calculated by normalizing plate displacement to the nominal initial hydrogel height of 4 mm. The apparent compressive Young’s modulus (herein referred to only as Young’s modulus) was determined from the slope of the linear region of the engineering stress–strain curve between 5–15% strain, excluding the initial toe region associated with plate seating/contact artifacts. Samples exhibiting visible tearing, slipping, or nonuniform deformation during compression were excluded from analysis. Mechanical properties were evaluated using n = 3–5 independent hydrogel replicates per formulation.

### 2.4 Cell Encapsulation and Growth Tracking in Hydrogels

For bacterial growth tracking, hydrogels encapsulating *B. subtilis* were formed at 100 μL volumes in 48-well plates and optical density at 600 nm (OD_600_) was monitored during culture using a microplate reader (Epoch2, Biotek). This experimental setup was designed to probe bacteria while confined in hydrogels with diffusive exchange of nutrients and metabolic products to and from the bulk fluid. These confinement and transport principles are shared across several biotherapeutic formats, including implantable or injectable bacteria-loaded hydrogels, skin and wound facing hydrogels.^40–42^ The 100 μL volumes were necessary to align with our 48-well plate reader and were sufficient for probing bacteria growth dynamics governed by the varied nanoscale environments created across the different hydrogel samples.

Prior to encapsulation, *B. subtilis* cells were cultured in TSB for 24 h (32 °C, 215 rpm). Cells were then spun in a centrifuge (20 min, 4400 rpm), spent media was removed, then the cells were resuspended in PBS (pH 8.0). To account for a 50% dilution when combined with the PEG precursors during encapsulation, cells were diluted to twice the desired concentration in ATGN-phosphate buffer. For example, for a hydrogel encapsulating cells at OD_600_ = 0.1, cells were first diluted in ATGN-phosphate buffer to OD_600_ = 0.2 before adding the PEGDA and PEGTT solutions. After encapsulation, hydrogels were gently washed with 1 mL of ultrapure water for 30 s to remove any planktonic or loosely attached cells. Hydrogels were deposited in 48-well plates by adding 50 μL of the *B. subtilis* cell suspension to the wells, then 22.4 μL of PEGDA (49 mM) followed by 27.6 μL of PEGTT (20 mM) solutions were added into the wells to initiate the cross-linking reaction. Solutions were incubated at room temperature for 30 min, a time sufficient for complete hydrogel formation.^30^ After hydrogel formation, 1 mL of M9 minimal media was dispensed on top of each hydrogel for culture, then bacterial growth was tracked over 72 h at 32 °C (unless otherwise stated), with OD_600_ measurements recorded every 10 min in all growth experiments. A 1 s linear shake at 567 cycles/min occurred before each measurement. M9 minimal media was used to create a well-controlled growth environment that enabled systematic investigation of bacteria growth behavior under different levels of hydrogel confinement. Growth kinetics were fitted to the Gompertz equation using Mathematica (Wolfram, Inc.), which models the bacterial growth curve as an asymmetrical sigmoidal function allowing for precise estimation of lag phase duration and growth rate.^43^ While the present studies focus on bacteria growth and behavior over shorter periods, these living hydrogel systems can be transitioned to function over longer periods through additional media supplementation or perfusion to sustain metabolically active bacteria.^40,44,45^

### 2.5 Surfactin Quantification in Cell Free Culture Fluid Using the CPC-BTB Assay

All surfactin quantification experiments were done using the cetylpyridinium chloride (CPC)-bromothymol blue (BTB) colorimetric assay from Yang et al., which was specifically developed to quantify surfactin concentrations in the cell free culture fluid (CFCF) of *B. subtilis* cultures and was validated against reverse-phase high-performance liquid chromatography (RP-HPLC).^46^ The CPC-BTB assay leverages the affinity of surfactin with CPC, dissociating the CPC-BTB complex to cause a color shift and a corresponding increase in absorbance at 600 nm. A 0.20 mM CPC solution was prepared by dissolving CPC in 0.1 M PBS (pH 8.0). Separately, a 0.20 mM BTB solution was prepared in the same buffer. Equal volumes of the CPC and BTB solutions were mixed in a glass tube and vortexed thoroughly to yield a uniform dark-green CPC–BTB indicator solution. This mixture was freshly prepared immediately prior to each assay. Culture fluid above each hydrogel after 48 or 72 h culture was then collected and centrifuged (4,400 × g, 20 min) to obtain CFCF, as any free cells can interfere with the CPC-BTB complex. For unconfined culture controls, CFCF was collected in the same manner after culture in identical medium containing the equivalent amount of nutrients and inoculated with the same initial number of bacteria. Then, 100 μL of CPC–BTB indicator solution was combined with 100 μL of CFCF in a 96-well microplate. The plate was agitated for 5 min in a microplate reader at 567 cycles·min^–1^ and orbital movement of 3 mm diameter to allow for CPC-surfactin complexation. Absorbance at 600 nm was then measured (Epoch2, Biotek). A schematic describing the surfactin quantification experiment can be found in the Supporting Information file (Figure S2). Surfactin concentration was quantified using standard calibration curves relating absolute surfactin concentrations (μg/mL) to absorbance at 600 nm. Surfactin standards were prepared using purified surfactin diluted in both M9 (Figure S3) and TSB (Figure S4) culture media to normalize for any background signal caused by the media components.

### 2.6 Fluorescent Imaging of Cells within Hydrogels after Encapsulation and Culture

An inverted fluorescence microscope (Nikon Ti-E, NIS-Element software) was used to image bacteria within hydrogels, both after encapsulation and at different stages of culture. For imaging, *B. subtilis* cells were encapsulated within a ∼25 μm thick hydrogel layer chemically attached onto 18 × 18 mm glass coverslips. Prior to hydrogel attachment, inert (fluorinated) glass slides (25 × 75 × 1 mm) and glass coverslips functionalized with a chemically reactive (thiolated) attachment layer were fabricated using a chemical vapor deposition method. For slide fabrication, each slide or coverslip was first cleaned by rinsing with 70% isopropanol in deionized water, rinsing with deionized water, and then drying with nitrogen. Cleaned slides or coverslips were then placed in a glass Petri dish (150 × 50 mm) next to an Eppendorf tube cap filled with 20 μL of either trichloro­(1H, 1H, 2H, 2H-perfluorooctyl)­silane for inert slides or (3-mercaptopropyl)­trimethoxysilane for thiolated coverslips. The glass Petri dish was set on a hot plate at 100 °C for 2 h. The hot plate was then turned off, and the slides/coverslips were left in the Petri dish overnight. Next, hydrogel precursor solutions containing *B. subtilis* 21332 cells were created as described in Section 2.4 at a 25 μL volume. Immediately after addition of the PEGTT, 7 μL of the precursor solution was pipetted onto a hydrophobic glass slide and placed onto a thiolated coverslip with 25 μm spacers separating the pieces of glass (Figure S5). This was incubated for 30 min to allow for hydrogel formation and chemical attachment to the thiolated coverslip. After gelation, the inert slide was peeled off, leaving the hydrogel attached to the thiolated coverslip. Before staining or culturing, each coverslip was washed 3× with ultrapure water. A detailed demonstration of hydrogel functionalization over glass coverslips is available in video format in Fattahi et al.^30^ The coverslip was placed in a 3D-printed coverslip holder (Figure S6A). The coverslip holder was designed in house; the STL file is available upon request.

To determine initial cell viability after hydrogel encapsulation, a Live/Dead Bacterial Viability Kit (Thermo-Fisher Scientific)^47^ was used. Here, hydrogels were stained according to the manufacturer’s instructions by adding 9 μL of the reagent mixture per 100 μL of hydrogel, followed by a 15 min incubation at room temperature in the dark. Excess dye was then removed by washing the samples with a 0.85 wt % NaCl solution. Fluorescence intensity was then recorded using FITC/TRITC filters at 10× magnification. The intensity ratio of green/red cells gives the overall initial cell viability using a calibration curve available in the Supporting Information file (Figure S1) in Gutierrez et al.^28^

For imaging cell growth within hydrogels during culture, hydrogel-functionalized coverslips (7 μL) were added to the coverslip holder which was then placed in a live cell incubation chamber (Tokai Hit, Figure S6B). 140 μL of a 50:50 mixture of M9 and ultrapure water was then pipetted on top of each hydrogel. To account for evaporation during the culture period, 67 μL of ultrapure water was added to the culture media every 24 h, which was equivalent to the amount of water evaporated. After the desired culture time, hydrogels were stained by pipetting 100 μL of a 10 μM SYTO 9 solution over the hydrogel and incubating for 15 min at room temperature in the dark. Hydrogels were finally washed three times with ultrapure water to remove excess or unbound dye and imaged (Figure S6C). All images were taken with a 10×, NA 0.3 objective and a FITC filter set.

### 2.7 Antibacterial Activity of *B. subtilis* Cell Free Culture Fluid

To assess the activity of surfactin produced by *B. subtilis*, 100 μL *H*_9.5_ hydrogels inoculated at 1 × 10^4^ cells per μL hydrogel were cultured with 1 mL of TSB at 37 °C for 48 h. The CFCF supernatant was then collected, centrifuged for 15 min at 4,400 rpm, and sterile filtered using a 0.2 μm filter to remove any residual cells. Surfactin levels in the CFCF were then quantified using the CPC-BTB assay calibration curve (Figure S4), then diluted to a final concentration of 1,000 μg/mL in fresh TSB media. This concentration corresponds to the reported minimum inhibitory concentration (MIC) for purified *B. subtilis* lipopeptide biosurfactant against *Escherichia coli*.^48^ 100 μL of the CFCF supernatant was then inoculated with *E. coli* at an initial OD_600_ of 0.1 in 96 well plates and cultured at 37 °C overnight. As a control, *E. coli* growth in supernatant from empty hydrogels without encapsulated *B. subtilis* cells was used under identical experimental conditions.

### 2.8 Preparation and Encapsulation of *B. subtilis* Spores

For experiments involving endospores, *B. subtilis* 21332 was first cultured overnight in TSB media (32 °C, 215 rpm), then centrifuged at 4,400 g for 20 min, washed twice with Milli-Q water, resuspended to OD_600_ = 0.1 in 2 × SG sporulation media and incubated in a shaker for 48 h (50 °C, 215 rpm). To purify endospores from any remaining vegetative cells, the culture tube was then placed in a 75 °C water bath for 20 min.^49^ To confirm the formation of endospores, the LIVE/DEAD BacLight Bacterial Viability Kit was applied according to the manufacture’s protocol, which stains spores or live cells green and dead cells red.^50^ 3 μL of each stain was added per 1 mL of bacterial suspension and incubated for 15 min in the dark at room temperature. A sporulation of ∼96% cells was quantified using image analysis. Spores were encapsulated within the hydrogel at an initial concentration of OD_600_ = 0.1 following the procedure described in Section 2.4. Following encapsulation, heat stress was applied to the hydrogels within the 48-well plate by placing it in a convection oven at 80 °C for 20 min. After heat treatment, the plate was cooled for 5 min then 1 mL of TSB was dispensed on top of each gel. Bacterial growth was then tracked for 48 h at 37 °C, with OD_600_ measurements recorded every 10 min. A 1 s linear shake at 567 cycles/min occurred before each measurement.

### 2.9 Statistical Analysis

SAS software (SAS Institute Inc.) was used for statistical analysis. Statistical significance was determined using a Student’s *t*-test and Tukey’s test, with an α < 0.05 considered statistically significant. All calculated means and standard deviations are based on a minimum of n = 3 independent replicates.

## 3 Results and Discussion

### 3.1 Generation and Characterization of Hydrogels of Varied Mesh Size

Molecular weights (MW) of both two-arm PEGDA and four-arm PEGTT macromers were first varied in precursor solutions to generate hydrogels with a range of average mesh sizes (ξ) (Figure 1). Equilibrium swelling theory^38^ calculations indicated that mesh sizes ranged from 9 to 19 nm with larger MW macromers generating larger mesh sizes, as expected. Statistical comparisons across all macromer combinations demonstrated that mesh size was tunable with PEG macromer MW (Figure 2A). The smallest mesh size (ξ_1_ = 9.5 ± 0.7 nm) was generated by PEGDA_2k_–PEGTT_5k_ macromers, the middle mesh size (ξ_2_ = 14.7 ± 0.8 nm) was generated by PEGDA_10k_–PEGTT_10k_ macromers, and the largest mesh size (ξ_3_ = 19.0 ± 0.6 nm) was generated by PEGDA_10k_–PEGTT_20k_ macromers. This mesh size range provides a distinct set of PEG hydrogels that can be fabricated reproducibly from commercially available PEG macromers and that are potentially suitable for ELM applications. The mesh size range is comparable to other step growth PEG hydrogels used in related investigations, including a 5–15 nm mesh size range created with PEG thiol-norbornene macromers for tunable protein release,^51^ and a 10–13 nm mesh size range created with PEG thiol–acrylate hydrogels for tunable hydrolytic degradation.^52^ While a ∼10 nm range in mesh size may seem small, when propagated throughout the entire hydrogel network, this range results in significantly different hydrogel characteristics, including notable macroscopic differences in swelling (Figure 2B). In fact, assuming a homogeneous network and cubic pores (as depicted in Figure 1), the difference from ξ = 9.5 to 19 nm results in a near order-of-magnitude difference in the number of pores (8×) in hydrogels of equivalent volumes. Hydrogels spanning this range of mesh sizes, herein referred to as *H*_9.5_, *H*_15_, and *H*_19_ to correspond with ξ = 9.5 nm, ξ = 15 nm and ξ = 19 nm respectively, were selected to enable systematic studies of bacteria function at varied levels of nanoconfinement. For clarity, Table 1 displays the notation for these hydrogels, as they are used in further studies.

**Figure 2:**
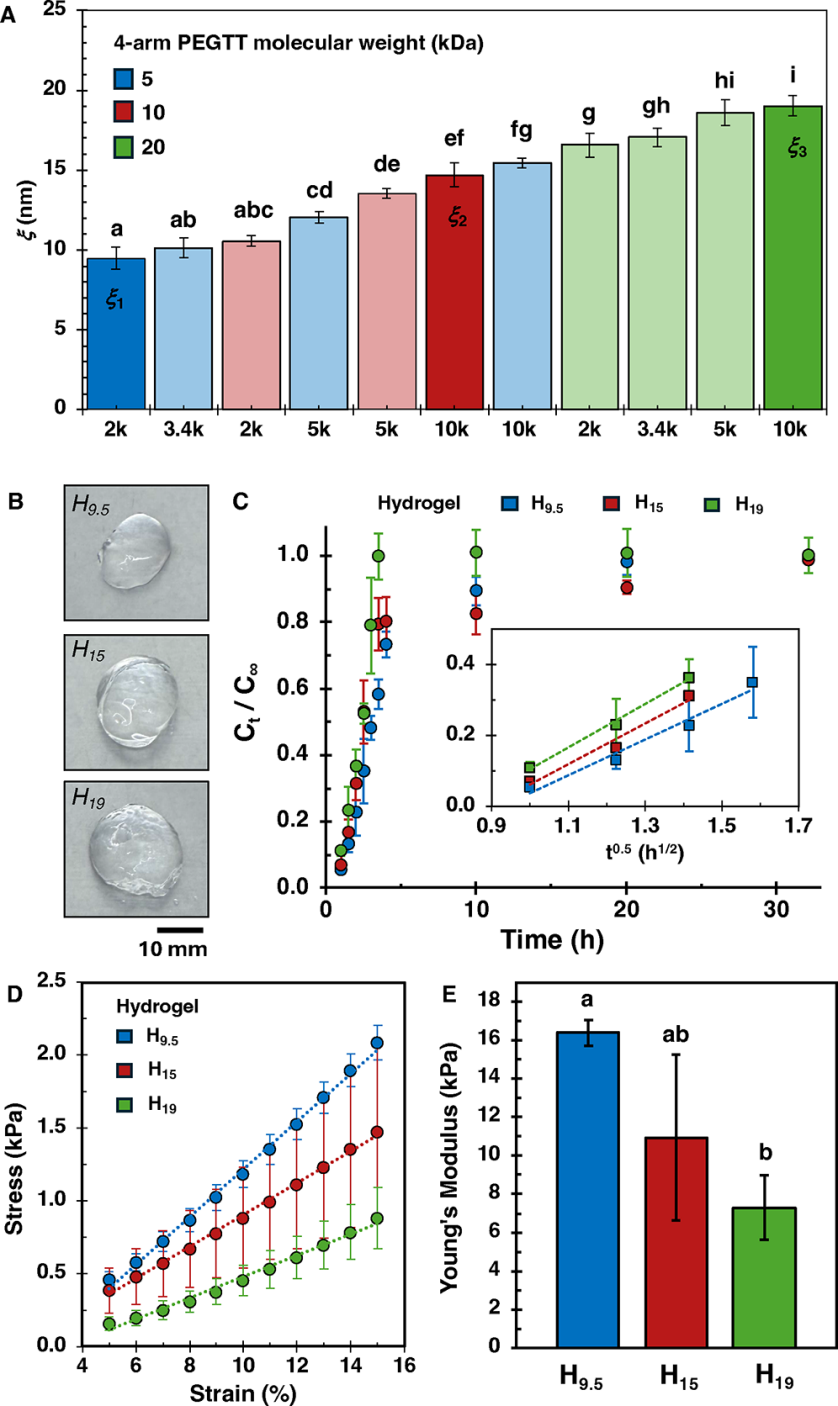
(A) Average mesh size (ξ) for each PEGDA and PEGTT macromer molecular weight combination. Error bars indicate standard deviation and differing letters indicate statistical significance (*p* < 0.05, *n* = 3). (B) Images of hydrogels of each mesh size chosen for further experiments after 24 h of swelling. All hydrogels were of equivalent 50 μL volumes prior to swelling. (C) Transient release of Alexa Fluor 488 from H_9.5_ (blue), H_15_ (red), and H_19_ (green) hydrogels. (Inset) Early time (C_t_/C_∞_ < 0.6) data used to determine D_eff_ a for Alexa Fluor 488 through the hydrogel. (D) Stress–strain curve for uniaxial compression tests and (E) Young’s modulus from the slope of the engineering stress–strain curve for H_9.5_, H_15_, and H_19_ hydrogels. Error bars indicate standard deviation (*n* = 3–5 replicates) and differing letters indicate significance (*p* < 0.05) by Tukey’s test.

**Table 1:** Notation of PEG Hydrogels for Cell Encapsulation at Varied Levels of Nanoconfinement^a^

Notation	PEGDA molecular weight (Da)	PEGTT molecular weight (Da)	ξ (nm)
*H*_*9.5*_	2,000	5,000	9.5 ± 0.7
*H*_*15*_	10,000	10,000	14.7 ± 0.8
*H*_*19*_	10,000	20,000	19.0 ± 0.6

a All hydrogels were prepared at 49 mM PEGDA and 20 mM PEGTT concentrations.

Following the quantification of average mesh sizes, mass transport through each of the three selected hydrogels was characterized. Here, diffusion of Alexa Fluor 488 through the hydrogel was measured, creating release profiles for each hydrogel (Figure 2C), Fick’s Law for early release (Figure 2C, inset) was then used to quantify diffusivity values of 5.17 ± 1.36 × 10^–7^ cm^2^/s, 1.04 ± 0.40 × 10^–6^ cm^2^/s, and 3.48 ± 0.47 × 10^–6^ cm^2^/s for *H*_*9.5*_, *H*_*15*_, and *H*_*19*_ hydrogels, respectively. For comparison, the diffusivity of Alexa Fluor 488 in water is 4.47 × 10^–6^ cm^2^/s when adjusted to room temperature,^53^ indicating that each hydrogel created diffusion limitations, and that diffusion became more hindered as mesh size decreased. Importantly, the 7-fold difference in diffusivity indicates these hydrogels created a wide range of nutrient environments for cell growth. Finally, hydrogel stiffness was quantified, as this is an additional factor that can influence bacteria growth in hydrogels.^54^ Stress–strain curves of *H*_*9.5*_, *H*_*15*_, and *H*_*19*_ hydrogels (Figure 2D) provide Young’s moduli values ranging from 16.4 ± 0.7 kPa to 7.3 ± 1.7 kPa (Figure 2E), falling in the expected range for PEG hydrogels with this mesh size range,^51^ and decreasing with increasing mesh size, as expected.

### 3.2 Cellular Growth in Hydrogels at Varied Levels of Nanoconfinement

After hydrogel characterization, a systematic study of *B. subtilis* growth and surfactin production was performed. Here, *B. subtilis* was cultured in M9 minimal media after encapsulation in hydrogels *H*_*9.5*_, *H*_*15*_, and *H*_*19*_ at a constant cell loading level (1 × 10^4^ cells per μL hydrogel). Images of *B. subtilis* cells throughout hydrogels immediately after encapsulation revealed differences in the initial distribution of cells, where the highest frequency of single cells and most uniform cell distributions were noted in *H*_*9.5*_ hydrogels (Figure S7A). As mesh size increased, larger cellular aggregates appeared more frequently, driving nonuniform cell distributions (Figures S7B and S7C). This may reflect the fact that higher molecular weight PEG precursors form hydrogels with more network heterogeneity,^51^ which could create larger local pockets that favor multicellular encapsulation and aggregation during hydrogel formation.

Cell microscopy at different growth stages (Figure 3A) and corresponding kinetic growth curves (Figure 3B) were used for comparative analysis across hydrogels. Growth within each hydrogel was marked by the disappearance of individual cells and formation of spherical aggregates (24 and 72 h, Figure 3A), and culture within each hydrogel displayed characteristic lag, exponential growth, and stationary phases (Figure 3B). Importantly, OD_600_ measurements were also taken from supernatant above each hydrogel (triangles, Figure 3B). These values remained consistently low during the culture period and did not increase over time, indicating that all hydrogel formulations retained encapsulated cells throughout the growth period. If cells had escaped from hydrogels and into bulk culture fluid, OD_600_ readings would have increased in the supernatant as it would become an unconfined culture.^28^

**Figure 3:**
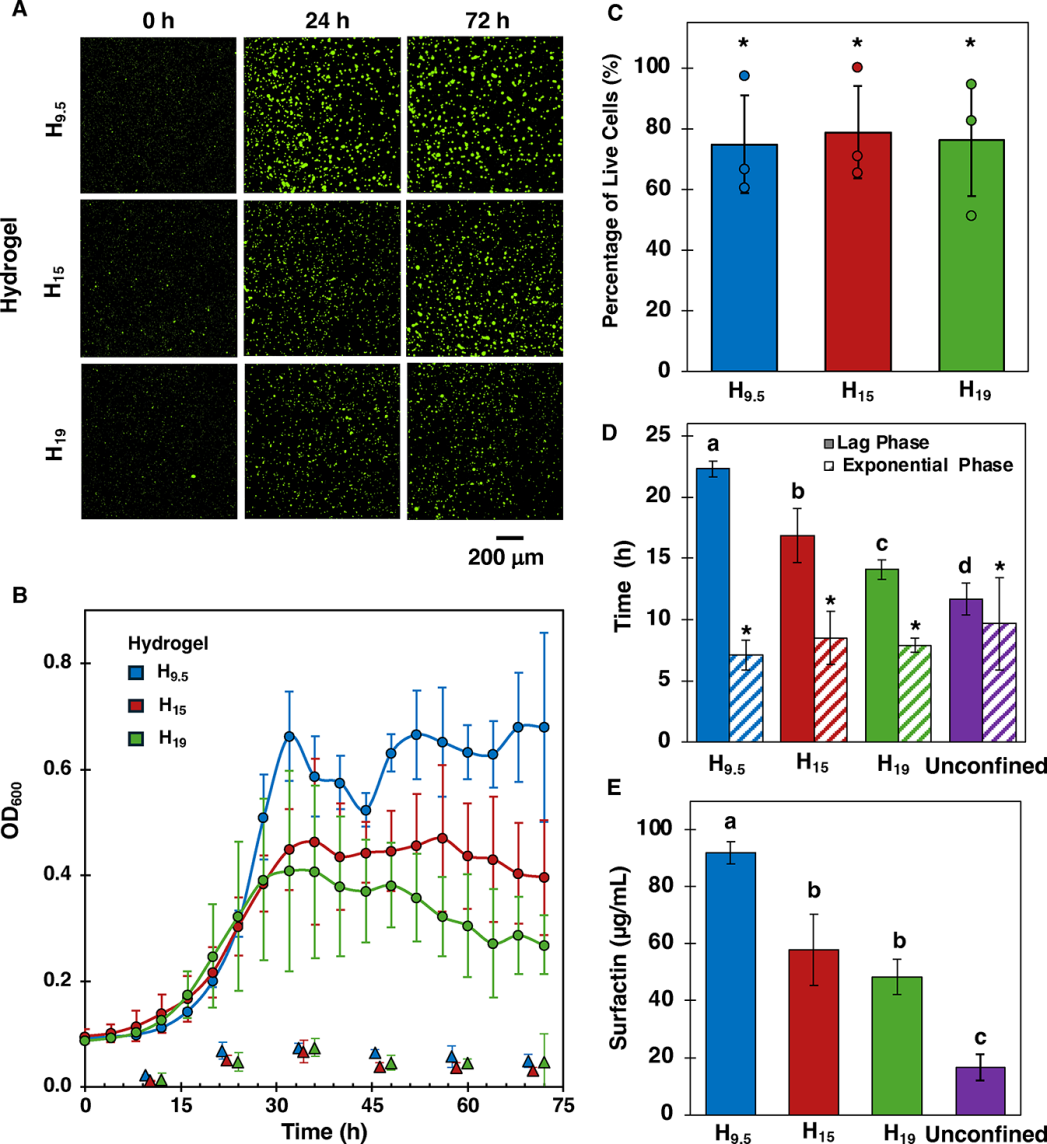
(A) 10× objective image montage of encapsulated *B. subtilis* 21332 at different culture stages for hydrogels of varied mesh size. (B) Growth kinetics of *B. subtilis* encapsulated within *H_9.5_* (blue), *H_15_* (red), and *H_19_* (green) hydrogels. Triangles: OD_600_ measurement of supernatant above encapsulated bacteria. (C) *B. subtilis* cell viability after encapsulation in selected hydrogels of varied mesh size. (D) Effect of hydrogel mesh size on lag (solid) and exponential (striped) growth phase duration. (E) Surfactin production from each hydrogel, measured using the CPC-BTB colorimetric assay. Error bars indicate standard deviation and differing letters indicate statistical significance (*p* < 0.05, *n* = 3). A single asterisk (*) is not significant, *p* > 0.05.

Noting that physicochemical hydrogel properties can impact bacteria viability,^4^ live–dead staining of *B. subtilis* immediately after encapsulation in each selected hydrogel was checked. Here, *B. subtilis* cells in *H*_*9.5*_, *H*_*15*_, and *H*_*19*_ had equivalent cell viability levels, indicating that mesh size did not significantly impact viability and that all formulations supported bacterial survival (Figure 3C). The averaged viability across all three hydrogels was 76.62 ± 16.6%, which is consistent with Gutierrez et al.,^28^ where PEGDA_3.4k_-PEGTT_10k_ (ξ = 12.3 ± 0.3 nm) hydrogels yielded a statistically equivalent level of cell viability (82.49 ± 12.36%).^28^ These findings are also consistent with analogous studies with mammalian cells by Zustiak et al.,^10^ where the viability of mouse fibroblast (Balb/3T3) cells after encapsulation in step growth PEG hydrogels of varied mesh sizes was characterized, and no correlation was found. This indicates that differences in initial cell viability after encapsulation was not a cause of growth differences between hydrogels.

Comparisons of growth curves (Figure 3B) within each hydrogel revealed significant differences. In particular, cells in *H*_*9.5*_ had delayed but more sustained growth to higher levels compared to *H*_*15*_ and *H*_*19*_, as indicated by OD_600_ values after 72 h. This agreed with corresponding microscopy images, where largest aggregates were observed in *H*_*9.5*_ hydrogels (20.5 ± 2.2 μm) at 72 h compared to 13.8 ± 1.4 μm and 11.6 ± 0.9 μm for *H*_*15*_ and *H*_*19*_, respectively (Figure 3A). To decipher differences in growth kinetics, the lag and exponential growth phases in each hydrogel were quantified (Figure 3D). For further comparison of growth to an unconfined culture, *B. subtilis* was also inoculated in bulk culture media under identical inoculum, growth media, and culture conditions, and its growth kinetics were also measured (Figure S8). A significant trend in the lag phase was found, as its duration increased as average mesh size decreased. No differences were found in the exponential phase. The lag phase in bulk culture was also significantly lower than the lag phases of all hydrogel cultures.

Taken together, these growth trends reflect the increasing diffusion limitations created with smaller mesh sizes, which when combined with the more uniform initial distribution of single cells, promoted more sustainable growth and larger aggregate formation. By comparison, more frequent aggregates and heterogeneous cell distributions created by larger pore sizes, accompanied with lower diffusion limitations and higher nutrient flux, led to faster local nutrient consumption around cell clusters to limit growth and eventually cause cellular decay.

### 3.3 Corresponding Surfactin Production in Hydrogels at Varied Levels of Nanoconfinement

Spatially confined environments can cause bacteria to coordinate gene expression and display cell density-dependent (i.e., quorum-mediated) behaviors, such as biofilm formation, chemotaxis, and secretion of metabolites.^55^ Further, artificial systems designed to confine cells within controlled micro and nanoscale environments, including microfluidic droplets^56^ and microwells,^57^ have been used to study quorum-sensing processes in a variety of bacteria. With respect to hydrogels, Gao et al. used *Vibrio harveyi* cells within alginate-based hydrogels to demonstrate that both the spatial distribution of cells and the mass transfer environment are critical components for bacterial quorum sensing.^58^ Their results indicate that both diffusion-limited environments and cellular aggregation within hydrogels promotes quorum sensing, and that larger aggregates produced higher levels of autoinducer molecules than smaller aggregates. Given this premise, and the fact that surfactin biosynthesis in *B. subtilis* is quorum-mediated,^59^ we hypothesized that *H*_*9.5*_, *H*_*15*_, and *H*_*19*_ would stimulate surfactin production according to the level of nanoconfinement.

For surfactin quantification, the CPC-BTB assay was used.^46^ The specificity of the CPC-BTB colorimetric assay to surfactin produced by *B. subtilis* CFCF was first validated by comparing the colorimetric response of surfactin-positive *B. subtilis* 21332 CFCF to surfactin-negative *B. subtilis* 168 (Δ*srfa* operon^60^) CFCF. The CFCF from *B. subtilis* 21332 caused a significant colorimetric shift and increase in absorbance at 600 nm, while *B. subtilis* 168 CFCF had no significant increase over background levels (Figure S9). Combined with RP-HPLC validation of surfactin specificity provided in Yang et al.,^46^ it was concluded that CPC-BTB assay could be used reliably for surfactin quantification from *B. subtilis* 21332 CFCF. Surfactin concentrations at 72 h for *H*_*9.5*_, *H*_*15*_, and *H*_*19*_ were next measured and compared to unconfined free-media culture prepared with identical inoculum, growth media, and culture conditions. The results revealed that hydrogel confinement was a strong promoter of surfactin production (Figure 3E), as a surfactin concentration of 91.8 ± 3.9 μg/mL was produced from *H*_*9.5*_ hydrogels, representing greater than a 5-fold increase relative to the unconfined culture, with a surfactin concentration of 16.6 ± 4.6 μg/mL. *H*_*15*_ and *H*_*19*_ hydrogels, with successively higher mesh sizes, showed corresponding decreases in surfactin levels, but were still significantly higher than the unconfined culture. Taken together with the growth measurements (Figure 3A–D), the results indicate the formation of larger aggregates and higher diffusion limitations created by smaller mesh sizes increase the quorum-mediated production of surfactin. As larger mesh sizes transition the hydrogel toward lower confinement and a more viscous liquid state, surfactin production approaches that of the unconfined culture.

In light of wound-healing and anticancer applications, it is notable that the surfactin concentrations measured from *H*_*9.5*_ to *H*_*19*_ in these experiments (48–92 μg/mL, Figure 3E) falls below the minimum inhibitory concentration (MIC) of most bacteria (ex. *Staphylococcus aureus*, 200 μg/mL;^27^ *E. coli*, 1,000 μg/mL)^48^ and below the levels reported to exhibit antitumor activity against cancer cell lines (100 μg/mL).^61^ However, this is due to the limited nutrients in the minimal M9 media used, and changing nutrient environment can increase surfactin production.^62^ To verify that similar gains in surfactin production could be obtained from hydrogel confinement in a more nutrient-rich environment, *B. subtilis* growth was again tracked in *H*_*9.5*_ hydrogels and unconfined cultures (Figure 4A and B) in TSB media, and corresponding surfactin levels after growth were again measured. A ∼4-fold gain in surfactin production was achieved with hydrogel confinement, where *H*_*9.5*_ hydrogels produced surfactin at a concentration of 1,841 ± 54 μg/mL (Figure 4C), which well exceeds concentrations necessary for antitumor activity and inhibitory concentrations of key pathogens in wound infections. To test the antibacterial activity of the solution released from the *B. subtilis*-PEG hydrogel, supernatant from these hydrogels was collected, centrifuged to obtain CFCF, then diluted to 1,000 μg/mL in TSB media to match the MIC for *E. coli* (Figure 4D).^48^ The growth of *E. coli* was then monitored during culture in 96-well plate format. As a control, supernatant from empty hydrogels with no *B. subtilis* was collected in the same manner and diluted in TSB media by the equivalent amount. Over a 20 h period, media supplemented with CFCF from the *B. subtilis*-PEG hydrogel diminished and eventually completely suppressed *E. coli* growth (Figure 4E). In stark contrast, *E. coli* proceeded to growth to high levels in the control media over the same period. This demonstrates the antibacterial activity of these hydrogels and advances its use toward biotherapeutic ELM applications that leverage bioactive secondary metabolites produced from hydrogels loaded with *B. subtilis*.

**Figure 4:**
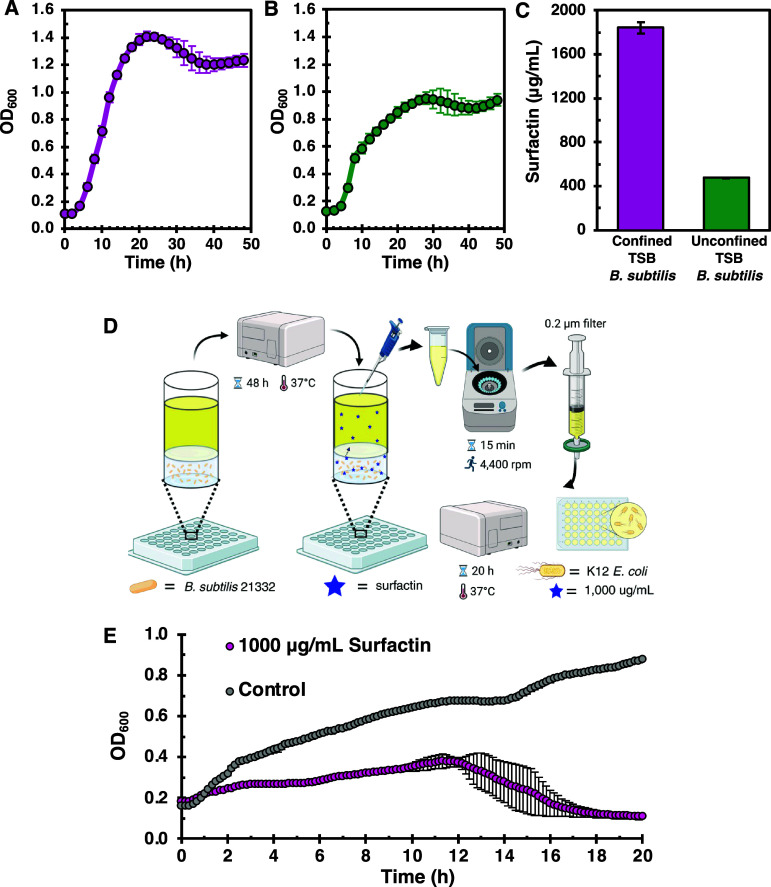
(A) Growth kinetics of *B. subtilis* during confinement within a *H_9.5_* hydrogel and (B) in unconfined culture. Both cultures used nutrient-rich TSB media. (C) Corresponding surfactin production in confined (magenta) or unconfined (teal) cultures. (D) BioRender sketch of experimental workflow for assessing bioactivity of hydrogel supernatant against *E. coli*. (E) Growth kinetics of *E. coli* inoculated with a fresh media-CFCF at 1,000 μg/mL surfactin concentration (magenta) and fresh media-supernatant from a gel with no encapsulated *B. subtilis* (control, gray). Error bars indicate standard deviation (*n* = 3).

### 3.4 Impact of Cell Loading on Surfactin Production

As *H*_*9.5*_ hydrogels produced the highest surfactin concentrations, these hydrogels were selected to next investigate the impact of bacteria cell loading, defined as the initial number of cells per volume of hydrogel precursor solution, on surfactin production. Cell loading was varied from 1.0 × 10^3^ to 7.5 × 10^4^ cells per μL hydrogel. These cell loading levels fall within a commonly reported range for other ELM bacteria encapsulation systems,^54^ including those used in wound-facing and injectable living hydrogels.^41^ After encapsulation at each cell loading level, equilibrium swelling experiments were again applied to determine if the presence of bacteria altered the average pore size. No significant difference in equilibrium swelling was found between the control set of hydrogels containing no bacteria and bacteria at any loading level (Figure S10), indicating that the presence of cells did not significantly alter the hydrogel network structure. Growth kinetics and surfactin concentrations in CFCF after 72 h culture were then measured in the same manner as previously described (Figure S2). Microscopic images of each hydrogel after encapsulation but before culture display a wide range of encapsulated cell densities with increased cell loading levels (Figure 5A), as expected. Prior reports from Rivera-Tarazona et al. have indicated that loading covalently cross-linked hydrogels with increasingly high cell loading levels can lead to the escape of cells from the hydrogel matrix.^63^ To check for cell release from the hydrogel during culture, the supernatant fluid was sampled after 72 h culture in M9 media for OD_600_ measurements, as done in the previous experiment. Low OD_600_ values were measured, indicating that cells were not released from the hydrogel at any cell loading level (Figure S11). At low cell loading levels (1.0 × 10^3^ to 1.0 × 10^4^ cells per μL hydrogel), cells within the hydrogels displayed growth trends according to the number of cells initially present (Figure 5B). Similar lag phases were measured, and OD_600_ values at 72 h increased with the initial number of cells loaded, indicating increased biomass accumulation within hydrogels as cell loading levels increased. Corresponding surfactin levels generated from this range of cell loading also increased with the number of cells loaded, specifically between 2.5 × 10^3^ to 1.0 × 10^4^ cells per μL hydrogel (Figure 5C). These trends indicate that at lower cell loading levels, cells initially encapsulated within the hydrogel were able to develop without interference from surrounding aggregates within the hydrogel.

**Figure 5:**
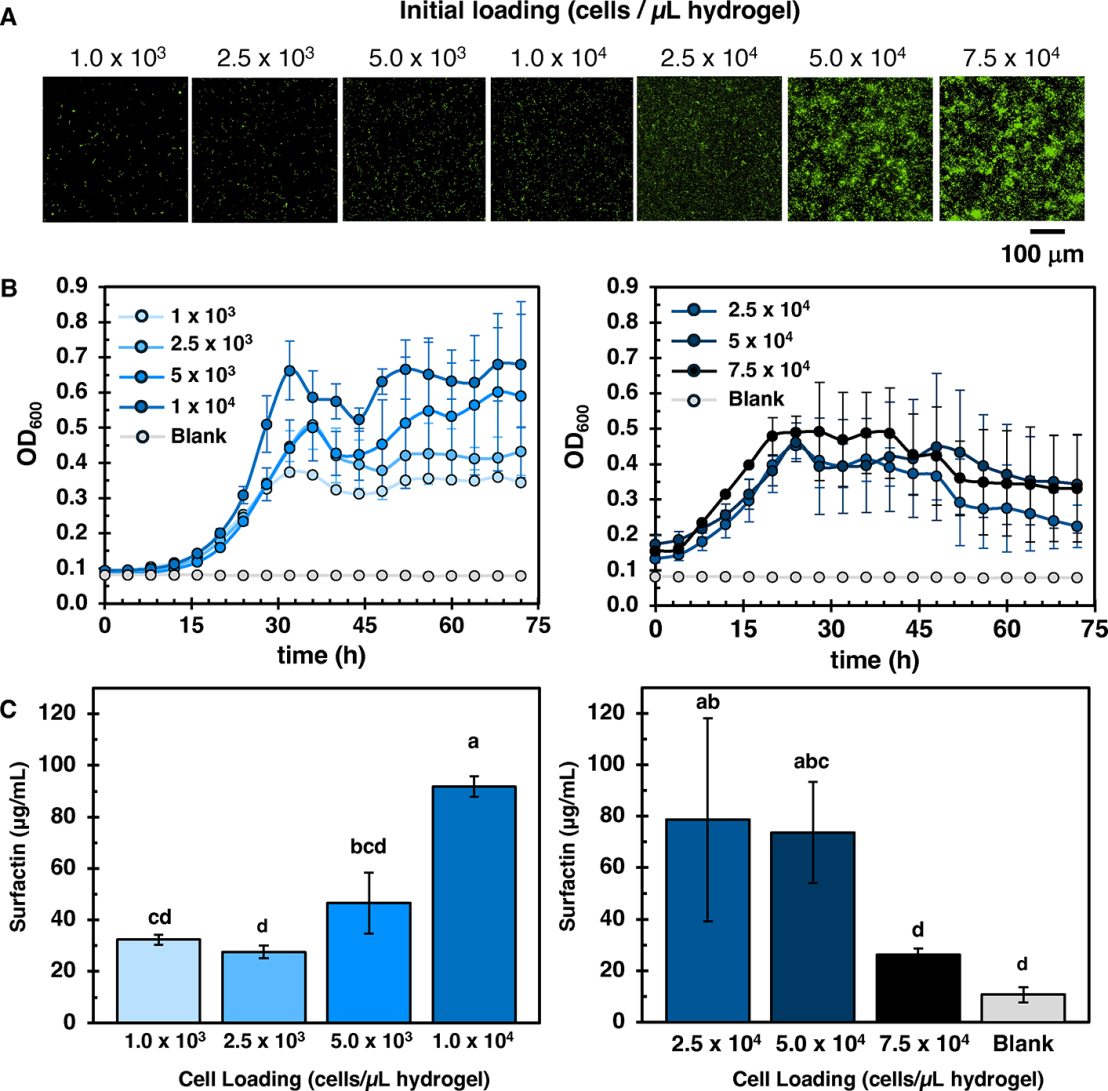
(A) 10× objective image montage of *H_9.5_* hydrogels with encapsulated *B. subtilis* 21332 at different initial cell loading densities (cells/μL hydrogel). (B) Growth kinetics of *B. subtilis* encapsulated within *H_9.5_* hydrogels at varied cell densities. (C) Corresponding surfactin production at 72 h. Error bars indicate standard deviation and differing letters indicate statistical significance (*p* < 0.05, *n* = 3).

Distinctly different behavior was observed when hydrogels were encapsulated with cells at higher loading levels (2.5 to 7.5 × 10^4^ cells per μL hydrogel). Here, growth kinetics were marked by a minimal lag phase and rapid growth phase to reach peak cell levels and the stationary phase after 24 h culture, followed by slow decay between 24 and 72 h (Figure 5B). Unlike the lower cell loading range, surfactin levels did not increase with cell densities at these higher loading levels. In fact, at the highest loading level (7.5 × 10^4^ cells per μL hydrogel), surfactin levels showed a significant decrease (Figure 5C). These trends indicate that loading hydrogels at high cell densities can cause interference between neighboring cell aggregates during culture. It was likely that nutrients within the culture media were rapidly consumed, leading to unsustainable growth and limited surfactin production. Similar observations have been made in other studies that investigate bacteria growth within spatially confined systems. For example, Hansen et al. seeded *P. aeruginosa* cells at different inoculum levels into microwells of subpicoliter volumes and monitored resulting cell growth during culture to determine that growth becomes inhibited as cells are seeded at excessively high inoculum levels.^64^ Taken together, these results demonstrate the importance of carefully determining optimal cell loading levels in accordance to the nutrient and physicochemical hydrogel environments; just as low cell densities can limit bioproduction, overloading cells within the hydrogel matrix can lead to interference between developing cellular aggregates to also limit or even inhibit bioproduction.

### 3.5 Encapsulation of *B. subtilis* Endospores for Surfactin Production after Temperature Stress

In nutrient-poor environments, *B. subtilis* can form dormant endospores with a multilayered architecture that preserves genomic integrity and enables long-term survival. By withstanding harsh environments common during ELM fabrication or storage, endospores hold decisive advantages for real world application. Previous work by González et al. demonstrated that *B. subtilis* endospores embedded within 3-D printed agarose hydrogels could be germinated to retain cellular function after exposure to desiccation, organic solvents, high acidity (pH = 1), ultraviolet and γ-irradiation, and high (75–100 °C) temperatures.^65^ However, prior studies have not investigated surfactin production from spore-laden hydrogels. This motivated the investigation of *B. subtilis* spores within *H*_*9.5*_ hydrogels, optimized from the prior studies, to achieve surfactin production after dehydration and exposure to elevated temperatures, conditions relevant for ELM storage.

Prior to hydrogel encapsulation, a live–dead assay was used to characterize the treatment of vegetative *B. subtilis* cells with 2 × SG sporulation media to obtain endospores. The live–dead stain was first performed on vegetative *B. subtilis* populations (no sporulation media) before and after treatment with a heating bath (75 °C, 20 min). Entire *B. subtilis* cell populations appeared live before the heat treatment and dead afterward (Figure 6A­(i) and (ii)), validating both the live–dead stain and the heat treatment protocol for removing live, vegetative cells. Next, populations of *B. subtilis* treated with 2 × SG sporulation media were also exposed to the heat bath. Live–dead staining indicated that 96 ± 1% of cells remained alive, with the remaining fraction of intact dead cells often appearing elongated (Figure 6A­(iii)). This indicates the successful formation of *B. subtilis* endospores using the 2 × SG sporulation media.

**Figure 6:**
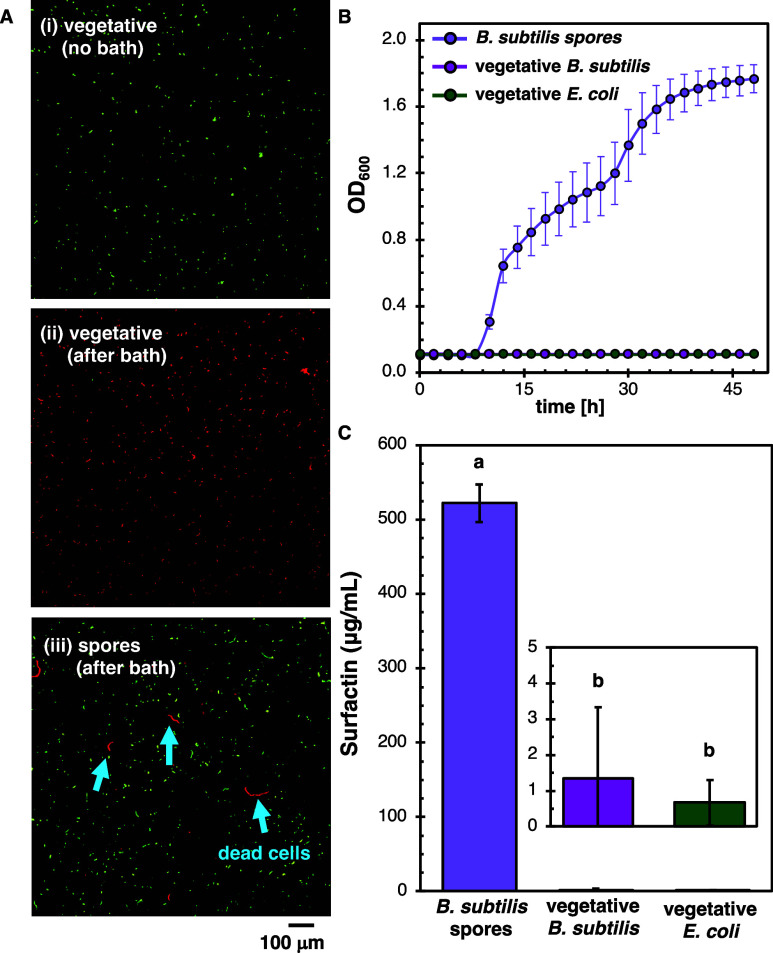
(A) 10× green-red fluorescent images of *B. subtilis* after live–dead staining of (i) vegetative cells before and (ii) after exposure to the water bath (75 °C, 20 min) and (iii) endospores after exposure to the same water bath. Green denotes live cells and red denotes dead cells, images were adjusted to maximize contrast in the figure. (B) Growth kinetics of encapsulated *B. subtilis* spores and *B. subtilis*/*E. coli* vegetative cells (negative controls) within *H_9.5_* hydrogels after double heat-treatment. (C) Surfactin quantification of *B. subtilis* endospores and *B. subtilis*/*E. coli* vegetative cells in *H_9.5_* hydrogels. Error bars indicate standard deviations and differing letters indicate statistical significance (*p* < 0.05, *n* = 3).

Following validation of the sporulation protocol, *B. subtilis* endospores were placed in a 75 °C water bath for purification, then encapsulated within *H*_*9.5*_ hydrogels at 1 × 10^4^ cells per μL hydrogel. To impose dehydration and temperature stress on the material, hydrogels were then exposed to a high temperature of 80 °C in an oven for 25 min, conditions that fully deactivate vegetative *B. subtilis*.^66^ Two negative control hydrogels were also performed. The first control used *H*_*9.5*_ hydrogels loaded with vegetative *B. subtilis* cells not treated with 2 × SG sporulation media, testing the ability of *B. subtilis* to adapt and survive the dehydration and temperature stress within the material without prior sporulation. The second control used *H*_*9.5*_ hydrogels loaded with *E. coli*, a Gram-negative, nonsporulating bacteria which grew as expected within hydrogels that were not heat-treated (Figure S12). All hydrogels were baked (80 °C, 25 min) then placed in TSB media, and growth kinetics and 48 h surfactin production of each were measured (Figure 6B and C), as in the previous studies. After an 8 h lag period, growth commenced only in hydrogels containing *B. subtilis* endospores, with a 48 h surfactin concentration of 522 ± 25 μg/mL (Figure 6C). The extended lag phase noted here is comparable to *B. subtilis* LMG16 spores exposed to temperatures ranging from 60 to 100 °C (Supp. Figure 28 in González et al.),^65^ and can be attributed to the time necessary to reactivate the spores. A second growth phase was observed after 28 h and could be due to cells being partially released into the growth media above the hydrogel, likely due to mechanical hydrogel fracturing of the hydrogel due to high bacteria proliferation in a nutrient rich media, a result also seen by Kalairaj et al.^67^ In contrast, no growth was measured in vegetative *B. subtilis* or *E. coli* cells after heat treatment (Figure 6B), and no significant amount of surfactin was measured (Figure 6C). The combined results indicate that the spore-laden hydrogel materials can produce surfactin after exposure to dehydration and heat stress, creating a more robust ELM material. While only short periods of environmental stress were investigated here, it is expected that these materials can survive similar stress for longer periods of time, as *B. subtilis* endospores can remain dormant indefinitely (i.e., millions of years).^68^

## 4 Conclusions

These studies provide three major findings that advance *B. subtilis*-PEG hydrogel systems toward ELM applications. First, hydrogel mesh size was found to be a regulator of microbial function, both with respect to growth and the amount of surfactin produced. Across all average mesh sizes examined (ξ = 9–19 nm), confinement within the hydrogel network boosted the production of surfactin compared to equivalent, unconfined cultures in bulk media. As low product yield and corresponding high production costs have traditionally limited surfactin production in *B. subtilis* bulk cultures,^69^ this finding uncovers a clear advantage of cellular nanoconfinement within hydrogels. The increased levels of surfactin production noted with decreasing mesh sizes was likely due to the more uniform initial distribution of single cells after encapsulation to drive the formation of larger cell aggregates, combined with the effects of increased nanoconfinement. The latter includes a stiffer hydrogel matrix and higher diffusion limitations—evidenced by the 7-fold drop in diffusivities measured across these hydrogels—to activate the quorum-sensing system in *B. subtilis* necessary for surfactin production. Second, cell loading studies demonstrated that while low cell densities result in limited surfactin production as expected, excessive cell loading also diminishes surfactin production due to unsustainable growth and reduced nutrient availability. This emphasizes the need for careful selection of cell loading levels in accordance with the nutrient and physicochemical environment to optimize material performance. Finally, *B. subtilis* endospores were loaded into hydrogels at optimized conditions to demonstrate production of surfactin after exposure to dehydration and heat stress. These fundamental insights lay the foundation for future design of PEG hydrogels in ELM applications that require stable encapsulation of bacteria, protection of bacteria from chemical and mechanical stresses, and optimized production of antimicrobial molecules or other secondary metabolites.

## Supplementary Material



## Data Availability

The data used in this study, the STL files used for the 3D printed coverslip holder and Young’s modulus measurements, the Mathematica code to calculate growth phases, and the macro used to calculate colony size in FIJI (ImageJ v2) are available upon reasonable request.

## Notes

Calculation of average mesh size using equilibrium swelling theory (S1.0); hydrogel length calculation for diffusion coefficient measurement (S2.0); summary of estimated hydrogel densities, initial masses, and swollen thicknesses (Table S1); images of 3D-printed materials for Young’s modulus measurement (Figure S1); experimental schematic of sampling the supernatant above the hydrogel to measure surfactin production (Figure S2); standard calibration curve of surfactin in M9 minimal media (Figure S3) and tryptic soy broth (Figure S4); overview image of setup used to create microscopically thin hydrogels (Figure S5); overview of 3D printed coverslip holder and setup for 72 h culture and subsequent imaging (Figure S6); cell aggregate histograms for *H*_*9.5*_, *H*_*15*_, and *H*_*19*_ (Figure S7); unconfined growth kinetics of *B. subtilis* 21332 in M9 minimal media (Figure S8); specificity test of the CPC-BTB colorimetric assay against *B. subtilis* 168 (Figure S9); equilibrium swelling ratios of varying initial *B. subtilis* cell densities (Figure S10); OD_600_ measurement of supernatant above hydrogels encapsulated with bacteria against blank controls with varying initial cell loading (Figure S11); growth kinetics of *E. coli* without heat treatment (Figure S12) (PDF)
