# Supplementary material for: PEG-Based Living Hydrogels Engineered for Tunable Production of Bioactive Lipopeptides
Source: ACS Appl Polym Mater. 2026 Jun 30;8(13):10210–22. doi: 10.1021/acsapm.6c00761 (PMC13366588; doi:10.1021/acsapm.6c00761)
Supplement: Supplementary file 1 [file ap6c00761_si_001.pdf]

SUPPORTING INFORMATION

PEG-Based Living Hydrogels Engineered for Tunable Production of Bioactive Lipopeptides

Jeffrey A. Reed[1], Moises M. Gutierrez[2], Douglas A. Dougherty[1], Asad Bin Zaman[1], Ryan R. Hansen[1,]*

[1]Tim Taylor Department of Chemical Engineering, Kansas State University, Manhattan, KS 66506, USA

[2]Department of Chemical and Material Engineering, New Mexico State University, Las Cruces, NM 88003, USA

[*]Corresponding author: Ryan R. Hansen, Address: 1017 Durland Hall, Manhattan, KS, 66506, United States of America. Email: rrhansen@ksu.edu

Page		Description
**S3**	**S1.0**	Calculation of average mesh size using equilibrium swelling theory
**S4**	**S2.0**	Hydrogel length calculation for diffusion coefficient measurement
**S6**	**Table S1**	Summary of estimated hydrogel densities, initial masses, and swollen thicknesses
**S7**	**Figure S1**	Images of 3D-printed materials for Young's modulus measurement
**S8**	**Figure S2**	Experimental schematic of supernatant taken to measure surfactin from hydrogels
**S9**	**Figure S3**	Standard calibration curve of purified surfactin dissolved in M9 minimal media
**S9**	**Figure S4**	Standard calibration curve of purified surfactin dissolved in tryptic soy broth (TSB)
**S10**	**Figure S5**	View of setup to create thin hydrogels on coverslips for microscopy
**S11**	**Figure S6**	Overview of 3D-printed coverslip holder and setup for 72 hour culture and imaging of hydrogels
**S12**	**Figure S7**	Cell aggregate histograms for $H_{9.5}$, H_{15}, and H_{19}
**S13**	**Figure S8**	Unconfined growth kinetics of *B. subtilis* 21332 in M9 minimal media
**S14**	**Figure S9**	Specificity test of the CPC-BTB colorimetric assay for distinguishing surfactin from non-surfactin producing *B. subtilis* strains
**S15**	**Figure S10**	Equilibrium swelling ratios of varying initial *B. subtilis* cell densities
**S16**	**Figure S11**	OD_{600} measurement of supernatant above hydrogels of varying initial cell loading
**S17**	**Figure S12**	Growth kinetics of *E. coli* without heat treatment
**S18**	**Supporting Information References**	

S1.0 Average mesh size calculation using equilibrium swelling theory[1]:

S1.1 Mass swelling ratio (Q_m):

$$Q_m = \frac{M_s}{M_d} \quad (1)$$

Where:

M_d = mass of dry hydrogel

M_s = mass of swollen hydrogel

Volumetric swelling ratio (Q_v):

$$Q_v = 1 + \frac{\rho_p}{\rho_s} * (Q_m - 1) \quad (2)$$

Where:

ρ_p = polymer density (1.125 g / cm^3)

ρ_s = solvent density (water, 1.000 g / cm^3)

S1.2 Polymer volume fraction (ν_2):

$$\nu_2 = {Q_v}^{-1} \quad (3)$$

S1.3 Molecular weight between crosslinks (M_c):

$$\frac{1}{M_c} = \frac{2}{M_n} - \frac{\bar{v}}{V_1} * \frac{\ln(1 - \nu_2) + \nu_2 + \chi\nu_2^2}{\nu_2^{1/3} - \frac{\nu_2}{2}} \quad (4)$$

Where:

M_n = average molecular weight of the un-crosslinked gel (molecular weight of 2-arm PEGDA plus 2 * (1/4) of the 4-arm PEGSH molecular weight). For example, for $PEGDA_{2k}$ – $PEGSH_{5k}$ the M_n would be 2,000 + 2 * (1,250) = 4,500

$\bar{v}$ = specific volume of the polymer ($\frac{\rho_s}{\rho_p}$, 0.8889)

V_1 = molar volume of the solvent (water, 18 cm^3 / mol)

χ = polymer-solvent interaction parameter (0.426 for PEG-water)[2, 3]

S1.4 Number of bonds in the crosslink (n):

$$n = 2 * \frac{M_c}{M_r} \quad (5)$$

Where M_r = molecular weight of the repeat unit (44 g/mol for PEG)

S1.5 Root-mean-square end-to-end distance of the polymer chain in the unperturbed state $(r_o^{1/2})^2$:

$$(r_o^{1/2})^2 = l\sqrt{C_n n} \ (6)$$

Where:

l is the average bond length (0.146 nm)[2]

C_n is the Flory characteristic ratio of the polymer (typically 4.0 for PEG)[4]

S1.6 Mesh size (ξ) calculation:

$$\xi\ (\text{nm}) = v_2^{-1/3} * (r_o^2)^{1/2} \ (7)$$

S2.0 Hydrogel length calculation for diffusion coefficient measurement:

S2.1 Estimation of initial gel mass

Hydrogel precursor densities were estimated from the weighted density contributions from each of precursor stock solutions. Although hydrogels were prepared from a nominal 200 µL precursor volume, formulation-specific densities were used to convert measured gel masses into volumes during equilibrium swelling calculations.

PEGDA and PEGSH stock solutions were prepared in water. Stock solution mass concentrations were estimated from the molecular weights given from the manufacturer specific to the polymer lot and the corresponding molar stock concentrations. For example:

$$49\text{ mM 10kDa PEGDA} * 11{,}000\ \frac{\text{g}}{\text{mol}} = 0.539\ \text{g/mL}$$

$$20\text{ mM 10kDa PEGSH} * 10{,}897\ \frac{\text{g}}{\text{mol}} = 0.2179\ \text{g/mL}$$

$$\rho_{gel} = \sum \frac{\rho_i * V_i}{V_{gel}} \ (8)$$

Where:

ρ_{gel} = specific density of the gel formulation

ρ_i = density of the precursor

V_i = volume of precursor added

V_{gel} = volume of the gel

Initial gel mass ($m_{gel,i}$) can then be calculated by:

$$m_{gel,i} = \rho_{gel} * V_{gel} \quad (9)$$

S2.2 Estimation of the swollen hydrogel volume

Equilibrium swollen masses were used from the average mesh size determination to estimate swollen hydrogel volumes and subsequent hydrogel thickness used for diffusion coefficient estimation.

$$Q_m = \frac{m_{gel,f}}{m_{gel,i}} \quad (10)$$

The swollen volume of the hydrogel was then calculated using:

$$V_{gel,f} = Q_v * V_{initial} \quad (11)$$

Where Q_v is calculated using equation 4.

S2.3 Hydrogel length estimation

The internal cross-sectional area of the cylindrical glass vials used to estimate the swollen hydrogel length. To calculate the glass jar inner diameter, 1 mL of tryptic soy broth (TSB) was added to an empty vial and the liquid height was measured from the internal vial bottom to the bottom of the liquid meniscus. The internal vial radius was calculated to be 0.4955 cm, corresponding to an internal diameter of 0.991 cm. The internal glass jar cross-sectional area ($A_{c,jar}$) was then calculated to be 0.771 cm^2. The length of swollen hydrogel ($L_{gel,f}$) can then be calculated by:

$$L_{gel,f} = \frac{V_{gel,f}}{A_{c,jar}} \quad (12)$$

Table S1. Summary of estimated hydrogel densities, initial masses, and swollen thicknesses

**Hydrogel formulation**	**Estimated formulation density (ρ_{gel}, g/mL)**	**Estimated initial hydrogel mass ($m_{gel,i}$, mg)**	**Estimated swollen thickness ($L_{gel,f}$, cm)**
2kDA/5kSH (~9.5 nm mesh)	1.006	201.2	0.520
10kDA/10kSH (~15 nm mesh)	1.020	204.0	0.889
10kDA/20kSH (~19 nm mesh)	1.026	205.2	1.259

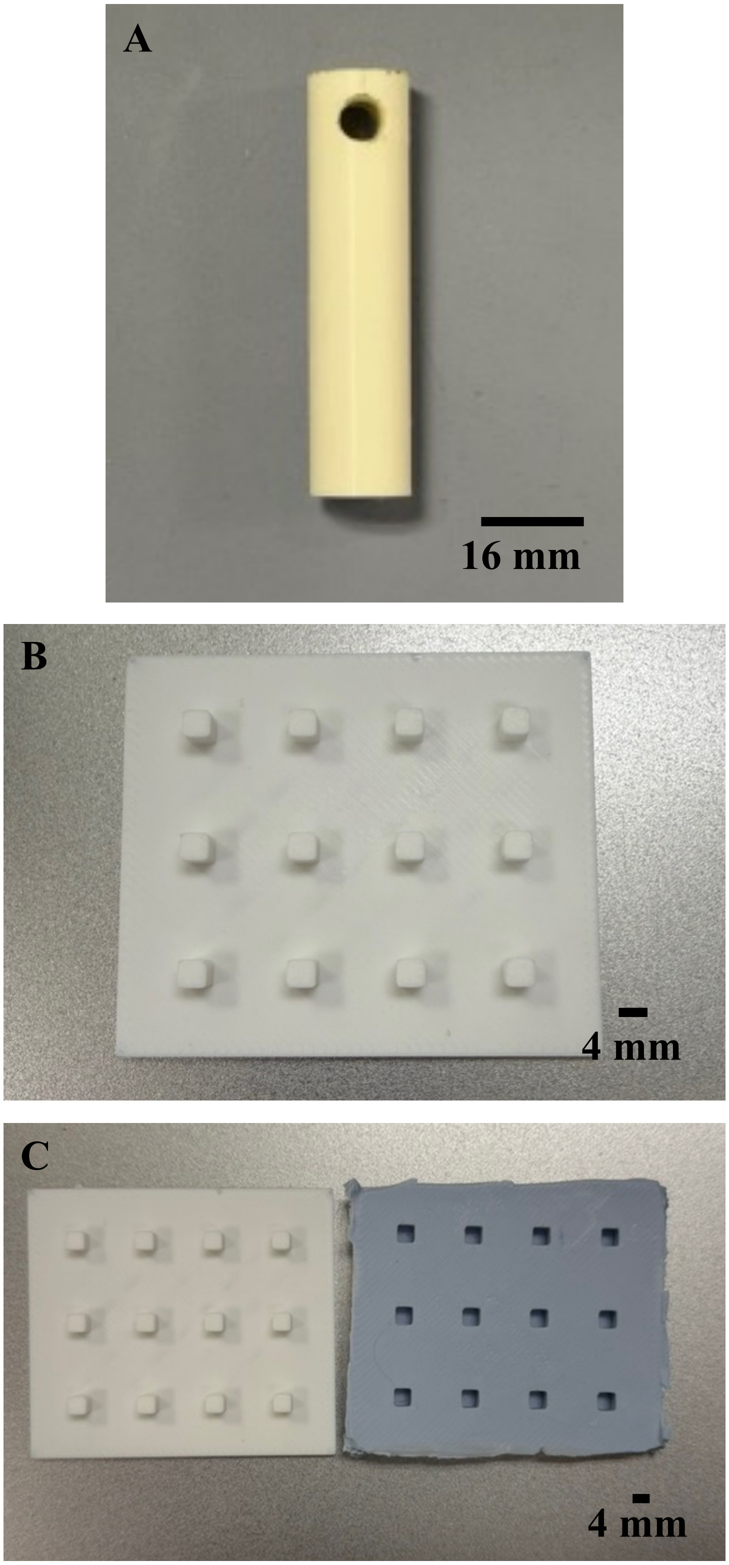

Figure S1. (A) Image of 3D-printed attachments to measure Young's modulus and (B and C) the plate/mold to create 4 mm hydrogel cubes.

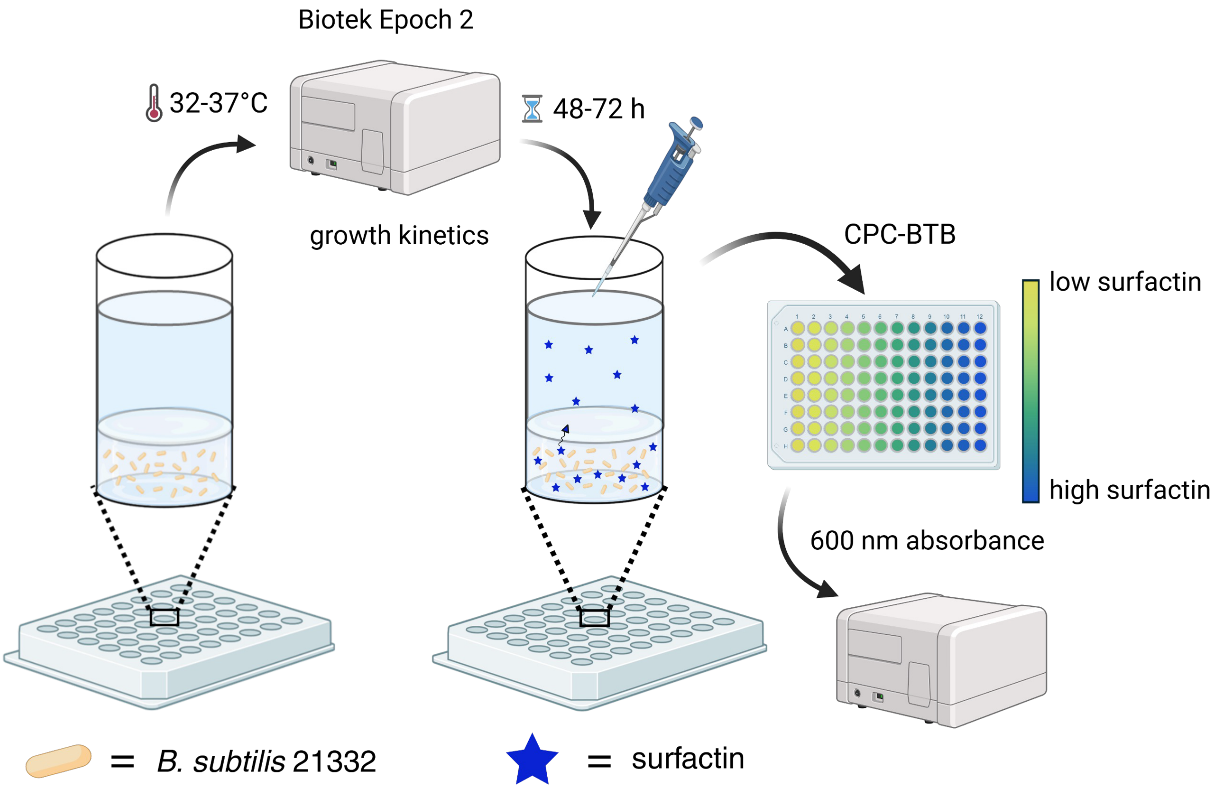

Figure S2. Experimental schematic of supernatant taken from above the hydrogel in 48-well plates to measure surfactin production from *B. subtilis* encapsulated in PEG hydrogels. Figure was completed using Biorender software.

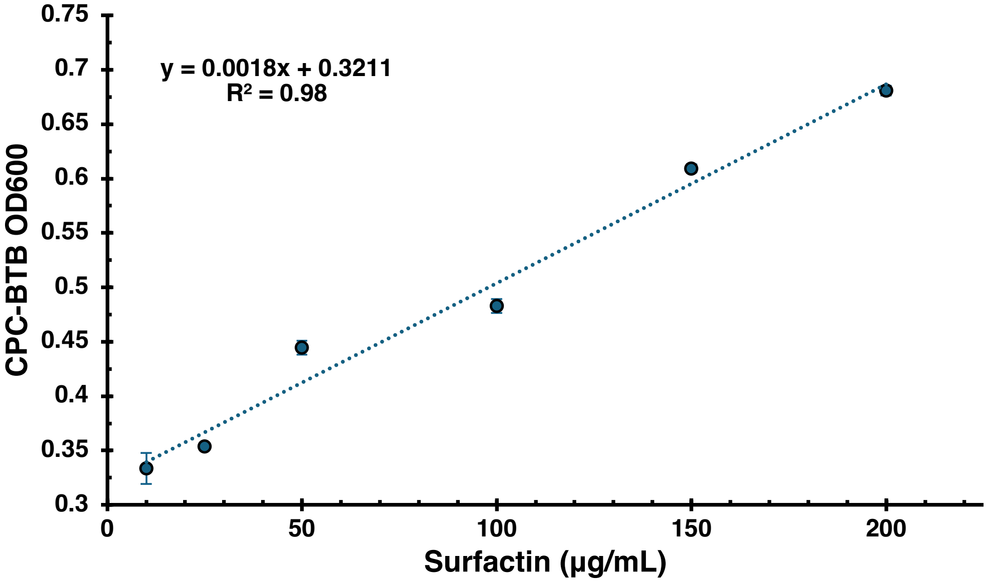

Figure S3. Standard calibration curve relating absolute surfactin concentration to absorbance at 600 nm using purified surfactin dissolved in M9 minimal media.

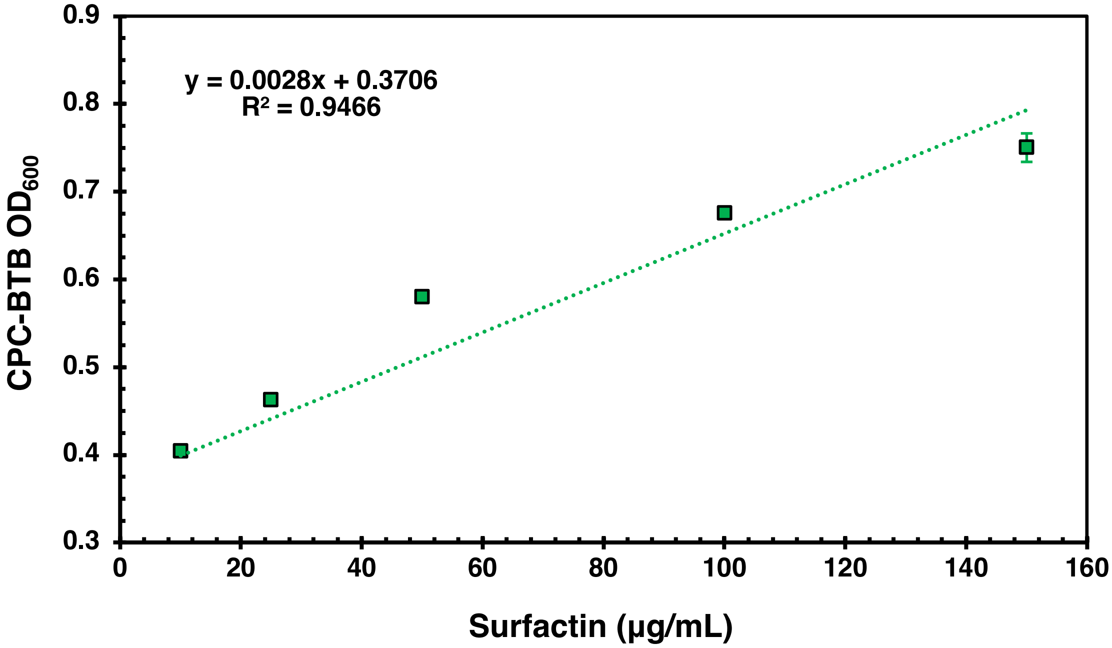

Figure S4. Standard calibration curve relating absolute surfactin concentration to absorbance at 600 nm using purified surfactin dissolved in tryptic soy broth (TSB) media.

A B

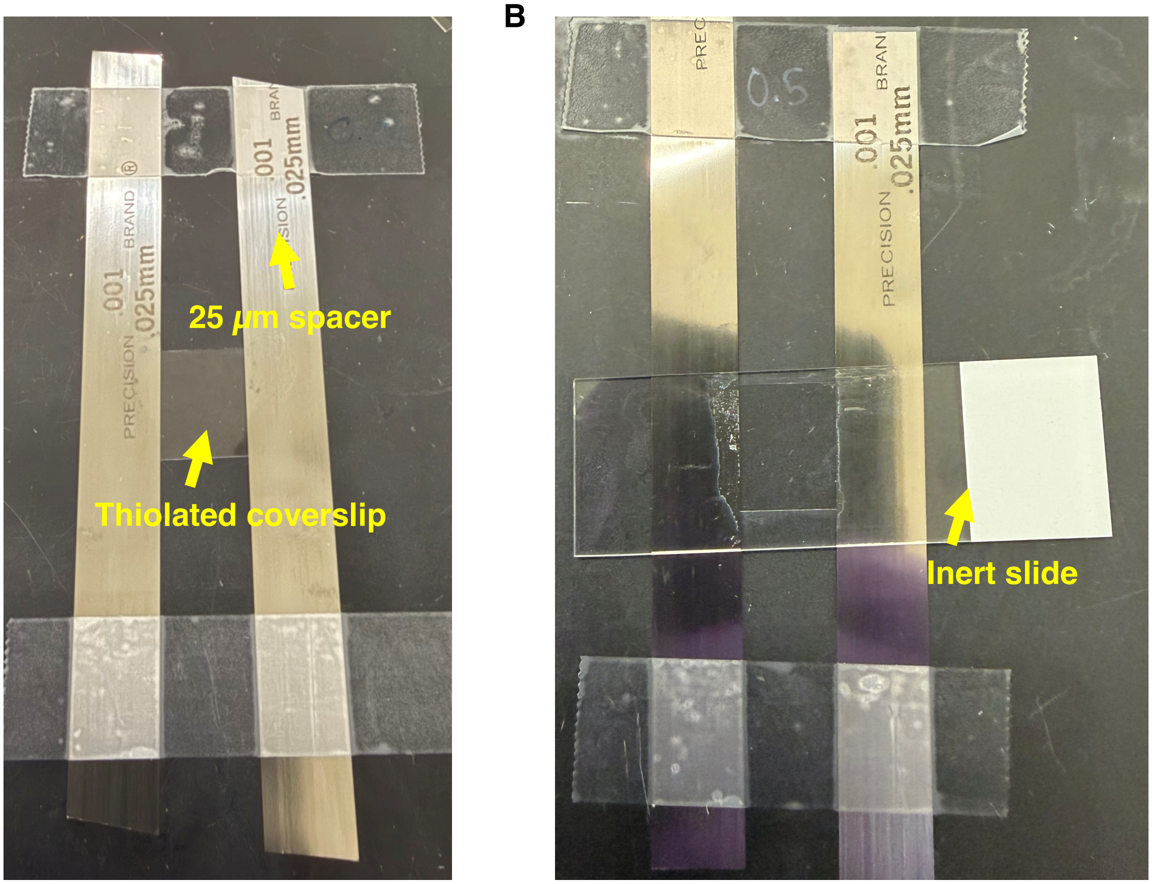

Figure S5. (A) Thiolated coverslip taped under 25 µm spacers to control hydrogel thickness. (B) hydrophobic/inert glass slide with hydrogel pressed onto the spacers.

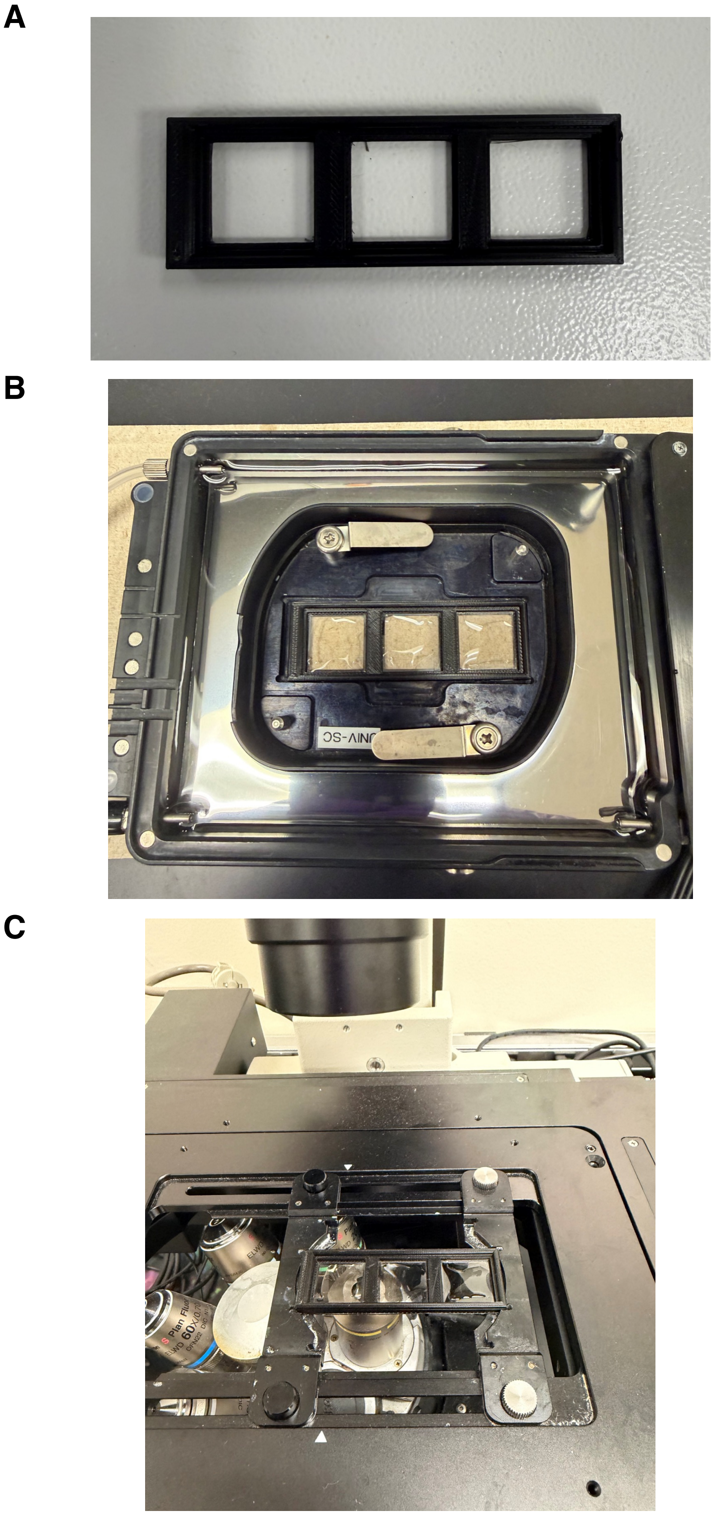

Figure S6. (A) 3D-printed coverslip holder to culture hydrogels on a coverslip. Each holder has room for three coverslips. (B) Coverslip holder inside the live cell incubation chamber. (C) View of imaging setup. The coverslip holder fits in the microscope stage allowing imaging without disturbing the hydrogels.

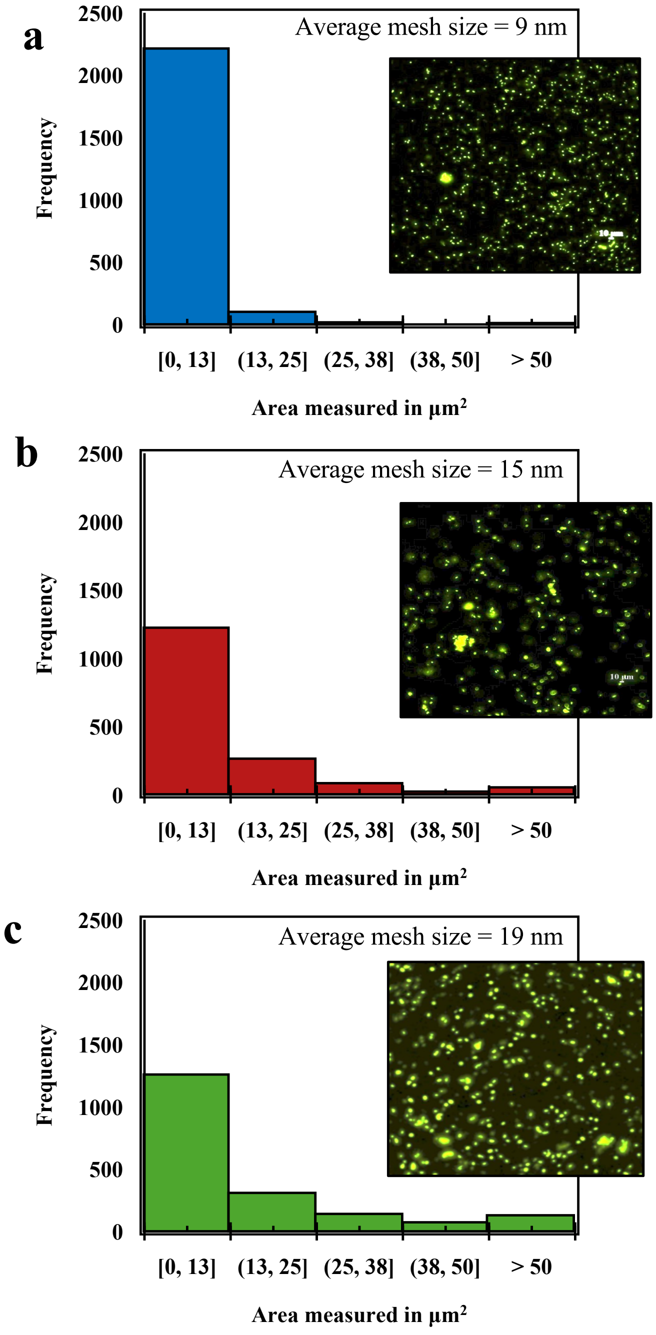

Figure S7. Effect of hydrogel mesh size on encapsulated *B. subtilis*-GFP from (a) PEGDA$_{2k}$–PEGTT$_{5k}$, (b) PEGDA$_{10k}$–PEGTT$_{10k}$, and (c) PEGDA$_{10k}$–PEGTT$_{20k}$ hydrogels as depicted by the histogram (left) and corresponding fluorescence microscopy (right). Data is based on (n=3) independent hydrogels for each mesh size.

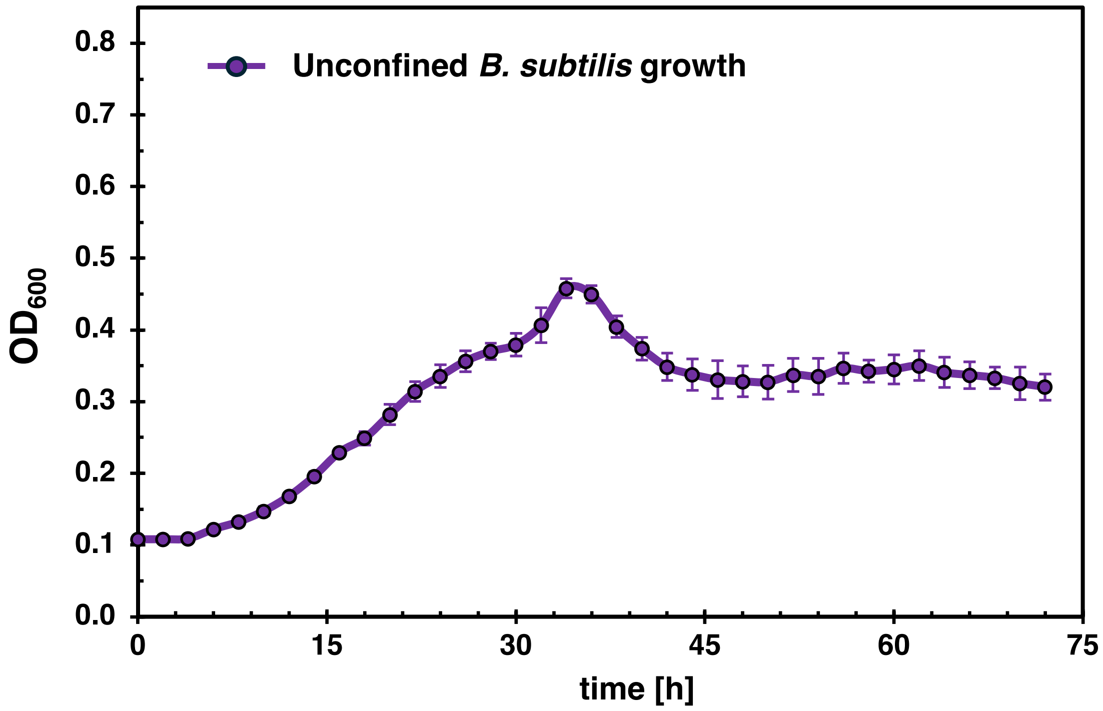

Fig. S8. Growth kinetics of unconfined *B. subtilis* in M9 minimal media culture under identical culture conditions as in confined hydrogels.

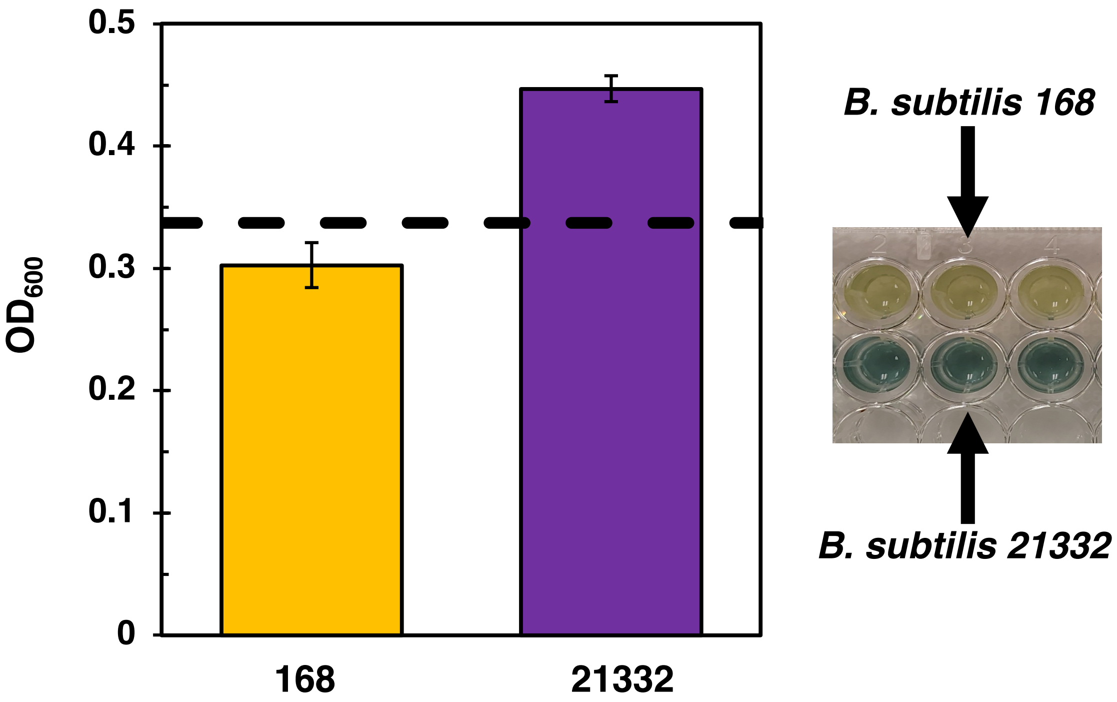

Figure S9. Specificity test of the CPC-BTB colorimetric assay comparing cell-free culture fluid of *B. subtilis* strain 168 (non-surfactin producing), *B. subtilis* strain 21332 (surfactin producing), and background (black dashed line)

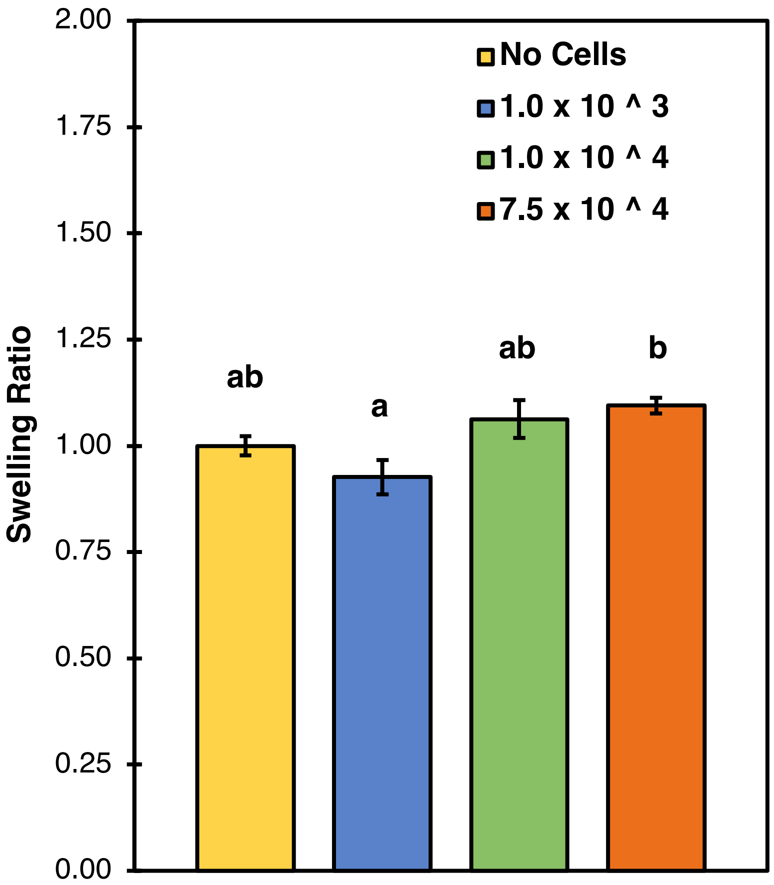

Figure S10. Equilibrium swelling ratios of $H_{9.5}$ hydrogels with low, medium, and high initial *B. subtilis* cell densities. Error bars indicate standard deviation (n=3) and differing letters indicate significance ($p < 0.05$) by Tukey's test.

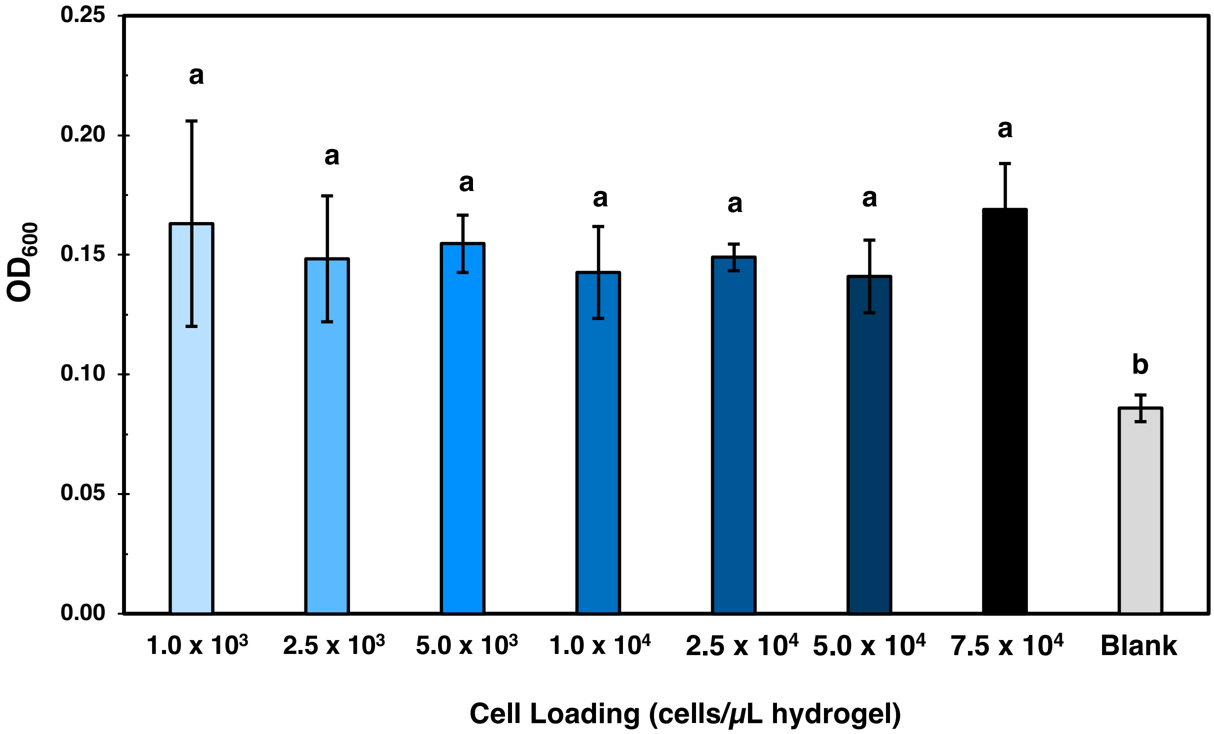

Figure S11. OD_{600} measurements of the culture media above the hydrogels after 72 hours for each initial cell loading inside the hydrogel. While all hydrogels with encapsulated bacteria had a small but reproducible and statistically significant increase in OD_{600} relative to blank controls ($p \leq 0.05$), this elevated signal is likely due to increased light scattering from extracellular material generated during culture, including cell debris, proteins, and extracellular polymeric substances released during cellular turnover over the 72 h culture period.

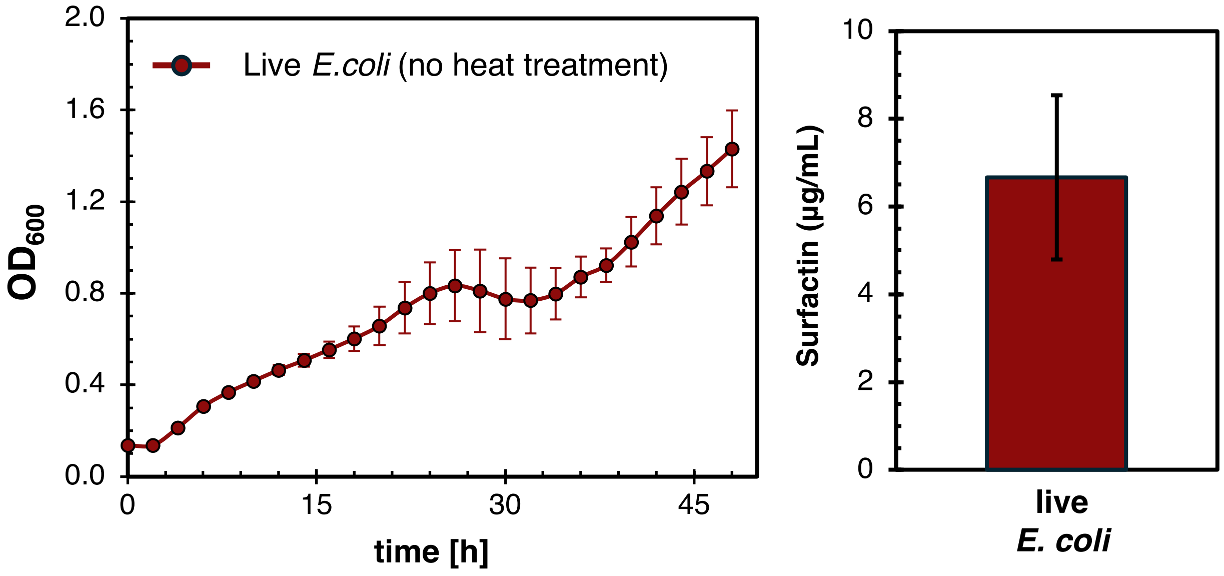

Figure S12. Growth (left) and surfactin measurement (right) of live *E. coli* encapsulated in H_1 hydrogels without heat treatment after 48 hours.

Supporting Information References

(1) Canal, T.; Peppas, N. A. Correlation between mesh size and equilibrium degree of swelling of polymeric networks. *J Biomed Mater Res* **1989**, *23* (10), 1183-1193. DOI: 10.1002/jbm.820231007 From NLM.
(2) Zustiak, S. P.; Leach, J. B. Hydrolytically Degradable Poly(Ethylene Glycol) Hydrogel Scaffolds with Tunable Degradation and Mechanical Properties. *Biomacromolecules* **2010**, *11* (5), 1348-1357. DOI: 10.1021/bm100137q.
(3) Cruise, G. M.; Scharp, D. S.; Hubbell, J. A. Characterization of permeability and network structure of interfacially photopolymerized poly(ethylene glycol) diacrylate hydrogels. *Biomaterials.* **1998**, *19* (14), 1287-1294. DOI: 10.1016/S0142-9612(98)00025-8.
(4) Rehmann, M. S.; Skeens, K. M.; Kharkar, P. M.; Ford, E. M.; Maverakis, E.; Lee, K. H.; Kloxin, A. M. Tuning and Predicting Mesh Size and Protein Release from Step Growth Hydrogels. *Biomacromolecules* **2017**, *18* (10), 3131-3142. DOI: 10.1021/acs.biomac.7b00781.
